# Deep Evolutionary Comparison of Gene Expression Identifies Parallel Recruitment of *Trans*-Factors in Two Independent Origins of C_4_ Photosynthesis

**DOI:** 10.1371/journal.pgen.1004365

**Published:** 2014-06-05

**Authors:** Sylvain Aubry, Steven Kelly, Britta M. C. Kümpers, Richard D. Smith-Unna, Julian M. Hibberd

**Affiliations:** 1 Department of Plant Sciences, University of Cambridge, Cambridge, United Kingdom; 2 Department of Plant Sciences, University of Oxford, Oxford, United Kingdom; Harvard University, United States of America

## Abstract

With at least 60 independent origins spanning monocotyledons and dicotyledons, the C_4_ photosynthetic pathway represents one of the most remarkable examples of convergent evolution. The recurrent evolution of this highly complex trait involving alterations to leaf anatomy, cell biology and biochemistry allows an increase in productivity by ∼50% in tropical and subtropical areas. The extent to which separate lineages of C_4_ plants use the same genetic networks to maintain C_4_ photosynthesis is unknown. We developed a new informatics framework to enable deep evolutionary comparison of gene expression in species lacking reference genomes. We exploited this to compare gene expression in species representing two independent C_4_ lineages (*Cleome gynandra* and *Zea mays*) whose last common ancestor diverged ∼140 million years ago. We define a cohort of 3,335 genes that represent conserved components of leaf and photosynthetic development in these species. Furthermore, we show that genes encoding proteins of the C_4_ cycle are recruited into networks defined by photosynthesis-related genes. Despite the wide evolutionary separation and independent origins of the C_4_ phenotype, we report that these species use homologous transcription factors to both induce C_4_ photosynthesis and to maintain the cell specific gene expression required for the pathway to operate. We define a core molecular signature associated with leaf and photosynthetic maturation that is likely shared by angiosperm species derived from the last common ancestor of the monocotyledons and dicotyledons. We show that deep evolutionary comparisons of gene expression can reveal novel insight into the molecular convergence of highly complex phenotypes and that parallel evolution of *trans*-factors underpins the repeated appearance of C_4_ photosynthesis. Thus, exploitation of extant natural variation associated with complex traits can be used to identify regulators. Moreover, the transcription factors that are shared by independent C_4_ lineages are key targets for engineering the C_4_ pathway into C_3_ crops such as rice.

## Author Summary

C_4_ photosynthesis is one of the most successful and widespread examples of convergent evolution; the first C_4_ plant evolved long after the extinction of the dinosaurs, yet C_4_ species now account for ∼30% of primary productivity on earth. Compared with ancestral C_3_ photosynthesis, the C_4_ pathway allows faster rates of growth, and thus international efforts have been mustered to introduce advantageous C_4_ traits into important C_3_ crops to increase their yield. However, the transition from C_3_ to C_4_ involves complex alterations to leaf anatomy and biochemistry. Despite these multiple changes, C_4_ photosynthesis has evolved independently at least 60 times. Through DNA and RNA sequencing we are beginning define a catalog of genes associated with C_3_ or C_4_ photosynthesis. However, we know little about how these genes act co-ordinately to bring about the convergent C_4_ phenotype. In this work we develop a new informatics framework to reveal that two independent lineages of C_4_ plants have co-opted the same regulators of gene expression to generate the C_4_ leaf. Our findings provide a new paradigm for investigating the genetics of convergent traits and the origin of convergent phenotypes. Moreover, they reveal significant new insight into the regulatory mechanisms governing the origins of C_4_ photosynthesis.

## Introduction

C_4_ photosynthesis is thought to have first evolved around 30 million years ago [1] and despite its complexity is now documented in more than 60 independent lineages of angiosperm [2]. Compared with ancestral C_3_ photosynthesis, the C_4_ pathway allows increased productivity in tropical and sub-tropical habitats, and C_4_ species represent many of the world's most productive crops [3]. The increased productivity of C_4_ plants is due to the fact that they concentrate CO_2_ around Ribulose Bisphosphate Carboxylase Oxygenase (RuBisCO) [4]. In most C_4_ species this is achieved through a spatial partitioning of the photosynthetic apparatus into two discrete cell types, mesophyll (M) and bundle sheath (BS) cells [4], [5], but in a small number of lineages spatial partitioning occurs within an individual cell [6], [7].

The entry point for CO_2_ in the canonical two-cell C_4_ pathway is *via* carbonic anhydrase (CA), which catalyses the conversion of CO_2_ to HCO_3_^−^ in M cells. Phospho*enol*pyruvate carboxylase (PEPC) utilizes HCO_3_^−^ to generate the C_4_ acid oxaloacetate and the subsequent diffusion of organic acids from M to BS cells, followed by their decarboxylation increases CO_2_ concentration around RuBisCO ten-fold [8]. This increase in CO_2_ concentration effectively abolishes the oxygenation reaction of RuBisCO and thus reduces energy loss through photorespiration. At least three different C_4_ acid decarboxylases (NAD-dependent malic enzyme, NADP-dependent malic enzyme and phospho*enol*pyruvate carboxykinase) have been recruited in different C_4_ lineages to release CO_2_ around RuBisCO in BS cells. To complete the canonical two-cell C_4_ cycle, phospho*enol*pyruvate is regenerated by pyruvate,orthophosphate dikinase (PPDK) in chloroplasts of M cells.

The patterns of gene expression that facilitate the compartmentalisation of photosynthesis between M and BS cells of C_4_ species have been assessed in a limited number of lineages. In dicotyledons, gene expression associated with maintenance of a functional C_4_ pathway has been studied in only two of the thirty-six known C_4_ lineages [9]. Moreover, in monocotyledons, the patterns of gene expression associated with generating a C_4_ leaf have so far only been reported in maize [10]–[13]. To date, several regulatory mechanisms have been demonstrated to play a role in modulating cell-type specific gene expression. These include both recruitment of *cis*-elements [14]–[17] and alterations to *trans*-factors [18]–[20]. While separate lineages of C_4_ species have co-opted the same *cis*-element to generate BS specific gene expression of NAD-dependent malic enzyme, the *trans*-factor is yet to be identified [18], and in fact, only one transcription factor known as *G2* has been shown to regulate photosynthesis gene expression in C_4_ leaves [20]. However, *G2* is not specific to C_4_ species and also regulates photosynthesis gene expression in C_3_ leaves [21]. Overall, these data indicate that the evolution of C_4_ photosynthesis is driven by both convergent and parallel changes in gene expression. However, it is unknown if these changes are governed by the same regulators.

Here we test the extent to which the same genetic networks regulate C_4_ photosynthetic development in independent lineages of C_4_ derived from the dicotyledons and monocotyledons. We defined a developmental gradient of C_4_ induction in the dicotyledon *Cleome gynandra* and characterised patterns of gene expression underlying this process. Currently, there are no C_4_ dicotyledons for which genome sequence is available, however analysis of *C. gynandra* is greatly facilitated by its phylogenetic proximity to the C_3_ model species *Arabidopsis thaliana* [9]. Through comparative analysis with an analogous developmental gradient in the distantly-related C_4_ monocotyledon maize [11], [12] we identify conserved sets of genes that underlie leaf maturation in both species. Although leaf maturation in monocotyledons is largely linear from base to tip [12], while in dicotyledons both basipetal and lateral gradients are apparent [22]–[24], we detected significant convergence in patterns of transcript abundance. We demonstrate that in both species genes important for the C_4_ cycle are co-regulated with photosynthesis-related genes and that eighteen transcription factor homologues form a common cohort underpinning C_4_ photosynthetic development in these species. We further report the degree to which M and BS transcriptomes overlap in *C. gynandra* and maize. Taken together, this work indicates that C_4_ photosynthesis is associated with the parallel evolution of *trans*-factors. This finding has major implications for engineering C_4_ photosynthesis into C_3_ crops such as rice [25] as it indicates that comparative analysis of multiple independent C_4_ lineages can facilitate the identification of the regulators underlying this complex trait.

## Results

### Immature leaves of *Cleome gynandra* develop mature C_4_ properties in a 3 mm interval

**Figure 1:**
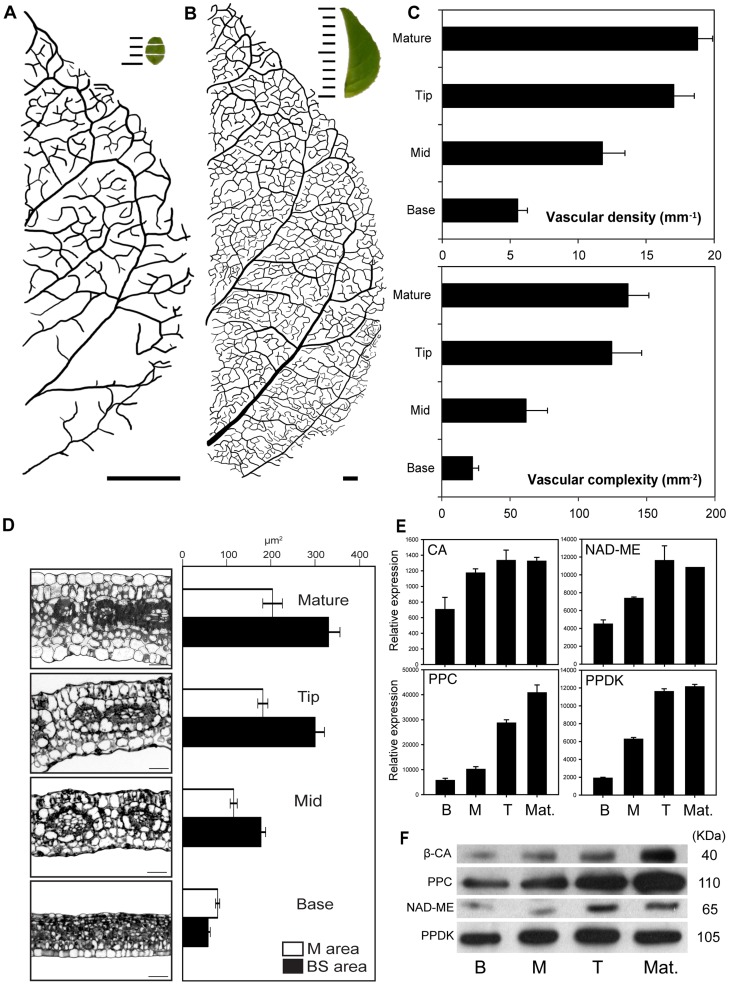
The C_4_ maturation gradient in leaves of *Cleome gynandra*. Venation, bundle sheath cell (BS) size, mesophyll (M) cell size and abundance of C_4_ transcripts and proteins in the base, middle and tip of 3 mm leaves as well as fully mature leaves of *C. gynandra*. (A) Leaves of 3 mm length possess a gradient in venation density from base to tip, whereas in mature leaves (B) this gradient is no longer visible, insets show representative images of samples used for RNA isolation. (C) Quantification of venation density and complexity. (D) Transverse sections and quantification of BS and M cell size. (E) Quantitative RT-PCR for the *CA4*, *PPC2*, *NAD-ME2* and *PPDK* of genes important in the C_4_ cycle. (F) Abundance of carbonic anhydrase, phospho*enol*pyruvate carboxylase, NAD-dependent malic enzyme and pyruvate,orthophosphate dikinase proteins from the base (B), middle (M), tip (T) and mature (Mat) leaves. Scale bars in A and B represent 0.3 mm and 3 mm respectively, while 1 mm gradations are shown within the insets.

Immature 3 mm long leaves of *C. gynandra* possessed gradients in Kranz anatomy, vein density and C_4_ gene expression from base to tip (Figure 1). Vascular density increased threefold (Figure 1A&B) achieving a density characteristic of mature leaves in the top third (tip) of 3 mm leaves (Figure 1B&C). The total cross section area occupied by mesophyll (M) and bundle sheath (BS) cells increased two- and six-fold respectively between base and tip sections. In the tip region of 3 mm leaves cell profiles were analogous to those seen in fully expanded mature leaves (Figure 1D). There were also pronounced differences in the rates of BS and M cell expansion between the base and middle section 3 mm leaves. The total BS cell area increased from 16% to 60% of the final size (3.8 fold increase), and the total M cell area only increased from 50% to 63% of the final size (1.3 fold increase, Figure 1D). Analogous gradients in maturation of cells, including increased chloroplast volume and vacuolisation, were also observed using transmission electron microscopy (Figure S1). The abundance of transcripts derived from key C_4_ genes [9] such as *CA4*, *PEPC*, *NADME2* and *PPDK* mirrored the increase in vascular density, with increases in abundance from base to tip of 3 mm leaves, but little difference between tip and mature leaf (Figure 1E). Similar increases in relative protein abundance for CA, PEPC, NAD-ME and PPDK proteins were also observed (Figure 1F). Together, these data demonstrate a progression of accumulation of key components for C_4_ photosynthesis from the base to the tip of 3 mm *C. gynandra* leaves. Moreover, the molecular and phenotypic signatures of the tip section appeared equivalent to mature leaves. Therefore, we exploited this framework to investigate patterns of gene expression underlying these phenotypic changes. Furthermore, we determined the extent to which these patterns of gene expression were analogous to those observed in the C_4_ monocotyledon maize [12]. To do this we sequenced RNA isolated from mature leaves as well as from consecutive 1 mm sections spanning these developing 3 mm leaves (Figure 1A), and implemented a novel bioinformatics framework that facilitates comparative analysis of gene expression in distantly related species.

### A novel machine learning method for orthology assignment of whole *de novo* assembled transcriptomes

To perform comparative analyses of gene expression between *C. gynandra* and maize it is necessary to be able to identify homologous genes between the species in the absence of a reference genome for *C. gynandra*. This is non-trivial due to the inherent properties and artefacts of *de novo* assembled transcriptomes. For example, it is to be expected that following *de novo* assemblies of RNAseq data, most gene loci will be represented by multiple assembled transcript variants [26]–[28]. These transcripts may differ from each other in several ways, for example through single nucleotide polymorphisms, alternative splicing of internal exons, alternative terminal exons and incomplete/chimeric assembly due to low sequence coverage or assembly errors. Homologous transcript identification is further complicated by the large phylogenetic distance between the species being compared. Increased phylogenetic distance leads to a concomitant increase in global sequence divergence between homologous genes in different species. Therefore any method which is specifically designed for assignment of homologues in *de novo* assembled transcriptomes should be able to identify and group multiple different transcript variants for any given gene to enable comparative analysis of gene expression.

**Figure 2:**
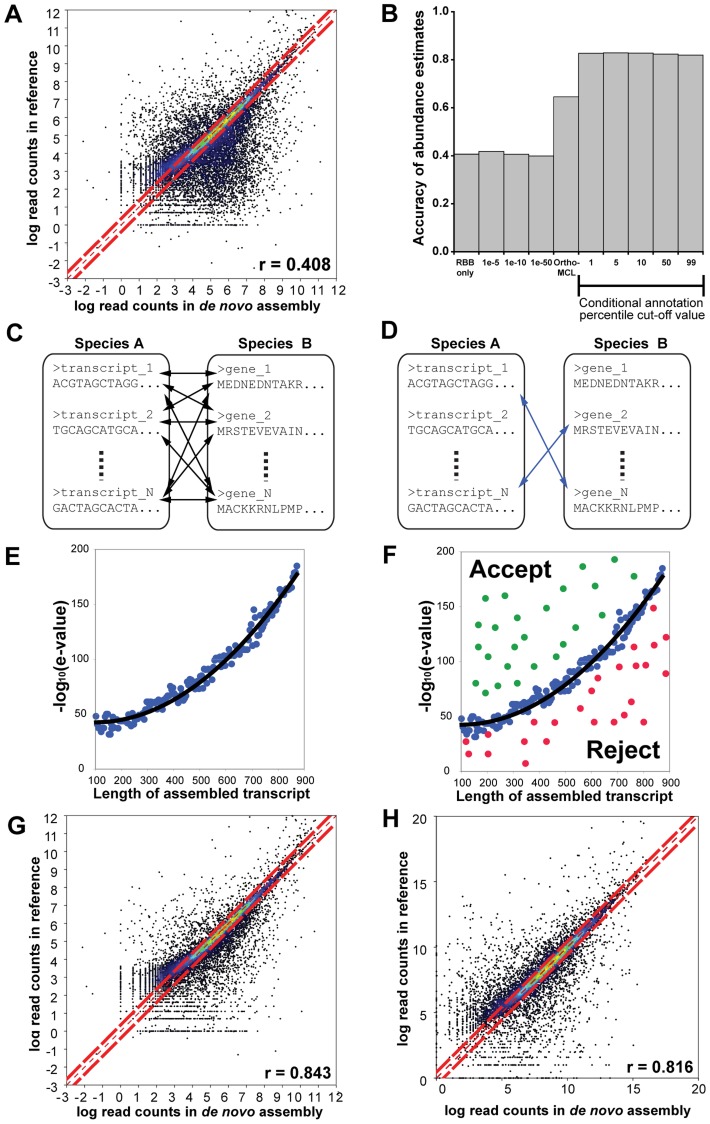
Overview of the workflow and results of the conditional orthology assignment method. Identification of homologues and quantification of gene expression after *de novo* assembly, for full details see Text S1. (A) Correlation in quantification derived from reciprocal best BLAST (RBB) hits in the *de novo* assembly and reference summed over all transcript isoforms per reference gene locus. (B) The Spearman correlation in transcript abundances between the reference guided estimation and estimates generated using different transcript orthology assignment methods on the same *de novo* assembled transcriptome. “RBB only” means that only the reciprocal best BLAST transcripts were selected. E-value cut-offs (e.g. 1e-5) indicate the fixed value at which sequences were determined to be homologues. OrthoMCL indicates that OrthoMCL was used to cluster and identify orthologous transcript groups. Finally, the black bar indicates the effect of varying the percentile cut-off on the abundance estimate accuracy of the conditional orthology assignment method. (C) Conditional orthology assignment method begins by performing all versus all BLAST searches of the assembled transcripts against a reference proteome. (D) The reciprocating hits (indicated by blue lines) are selected for self-training. (E) The reciprocating hits are binned according to assembled transcript length and a quadratic model is fit to the e-value and length data. (F) Non-reciprocating hits which fall above the curve are accepted as putative homologues, non-reciprocating hits which fall below the curve are rejected. (G) Correlation in quantification derived from conditional assigned transcripts using species own reference genome. (H) Correlation in quantification derived from conditional assigned transcripts using intermediary reference genome. For full details, validation and explanation please see the supplementary methods (Text S1).

To determine the suitability of existing assignment methods for identifying homologous transcript groups in *de novo* assembled transcriptomes we used RNAseq data from rice. Here we carried out *de novo* assembly of the short read data, and computed an abundance estimate for each *de novo* assembled transcript. We also computed an abundance estimate for each gene locus in the rice reference genome using the same short read data. Several different strategies for identifying homologous transcripts between the *de novo* assembled transcriptome and the rice reference genome were tested and the accuracy of each strategy was assessed by the global correlation of the abundance estimates that resulted from the assembled transcripts-to-reference-gene homology map. Global correlation is negatively affected both by false positive errors (incorrect homology assignment), false negative errors (missing orthology assignment) and assembly artefacts (partial and chimeric transcripts) and so it is a good measure of the utility of an orthology assignment method for quantitative transcriptome comparisons. When using simple methods such as a Reciprocal Best-BLAST (RBB) or fixed e-value cut-offs for assignment abundance estimate accuracies were low and unsuitable for comparative gene expression analyses (Figure 2A & 2B). Using more complex methods such as OrthoMCL improved abundance estimate accuracy (Figure 2B). However accuracy is still low for comparative analyses of gene expression.

The abundance estimate accuracy tests revealed that there was room for substantial improvement of orthology assignment from *de novo* assembled transcriptomes. As there are no specific methods currently available which are designed to account for the properties and artefacts of *de novo* assembled transcriptomes as outlined above, we developed a novel orthology assignment method to facilitate accurate multispecies comparisons of gene expression from *de novo* transcriptome assemblies. The method uses machine learning to define sequence similarity parameters for gene homologues and thus compensates for the properties and artefacts of *de novo* assembled transcriptomes. The first step in this method is to undertake a pairwise reciprocal best-BLAST (RBB) analysis (Figure 2C) using the full set of *de novo* assembled transcripts against a reference set derived from a reference genome. The RBB hits between these two datasets are identified (Figure 2D) and grouped according to the length of the assembled transcript. For each length group the RBB hits are ranked and the e-value of a chosen percentile is recorded. A matrix of all e-values and query sequence lengths is then fit to a quadratic polynomial model by least-squares fitting (Figure 2E). While the RBBs are accepted as homologues, the function describing this curve is used to classify non-RBB transcripts of any given length, those above the curve are assigned as homologues and those below the curve are rejected (Figure 2F). Thus homologue assignment is conditioned on both the assembled transcript length and also the global sequence divergence between the *de novo* assembled and reference transcriptome. This approach significantly increased the accuracy of abundance estimates derived from *de novo* assembled transcripts when compared with estimates derived from the genome (Figure 2B and 2G). This accuracy is also robust to large phylogenetic distances. Even when homologous transcripts were identified using an intermediary reference genome (*Arabidopsis thaliana*), the accuracy of mRNA abundance estimates remained high (Figure 2H). We conclude our assignment method, conditioned on both sequence length and global sequence divergence, is suitable for comparative analyses of gene expression after *de novo* transcript assembly from short read sequencing. For a detailed description and validation of this method see Text S1. This approach is also suitable for identifying homologous groups in distantly-related species (see Text S1 for validation on *Oryza sativa* versus *A. thaliana*). Thus we used this method to enable comparison of gene expression between *Cleome gynandra* and maize, an equivalent phylogenetic distance. An online implementation of the method is provided for use at www.bioinformatics.plants.ox.ac.uk/annotate/index.html.

### Transcriptome dynamics during *C. gynandra* leaf development

**Figure 3:**
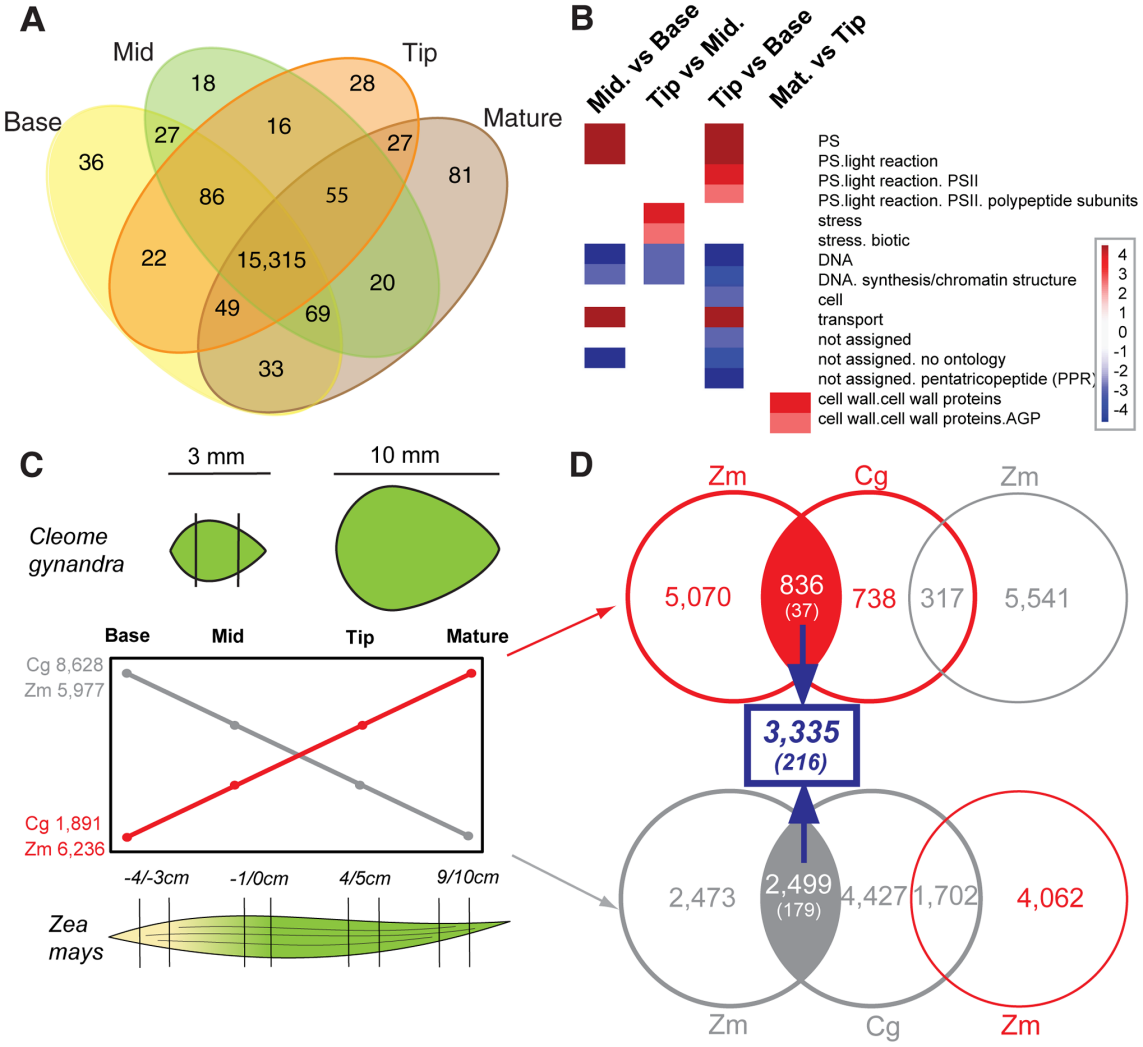
Convergence in patterns of gene expression in leaf gradients of *C. gynandra* and maize. (A) Venn diagram indicating numbers of shared and unique transcripts to each type of *C. gynandra* leaf tissue. (B) Major bin categories identified using Wilcoxon test implemented in Pageman [55] tool that alter between the base, middle, tip of 3 mm and mature *C. gynandra* leaves. (C) Number of genes with ascending (red) and descending (grey) behaviours as leaves of *C. gynandra* (Cg) and maize (Zm) mature. (D) Venn diagrams depicting the total number of transcript homologues that increase or decrease in abundance as leaves of both *C. gynandra* and maize mature. The number of genes common to the two gradients is shown in blue, with the number of transcription factors shown in parentheses. Red circles and numbers correspond to genes that increase in abundance, while grey circles represent genes that show reduced abundance.

Following *de novo* assembly, we used our orthology assignment method to assign all observed transcripts to reference genes in the genome of *A. thaliana*. *A. thaliana* was selected as it is the closest relative of *C. gynandra* for which a well annotated set of genes and gene models is available. This resulted in the identification of 15,751 genes of which 15,315 (97%) were expressed in all *C. gynandra* samples (Figure 3A). 36 genes were expressed only in the base of developing 3 mm leaves, compared with 18 and 28 in the middle and tip respectively, while there were 81 genes expressed only in mature leaves (Figure 3A). The higher number of genes specific to the leaf base compared with the middle and tip likely reflects the earlier stage of development of this tissue. Consistent with this, the majority of gene annotations in this subset comprise regulatory functions such as gene expression, translation and signalling (Table S1). Genes unique to the middle section of developing leaves were fewer in number and were mostly annotated as being involved in DNA binding, gene expression, protein binding or having unknown functions (Table S1). Comparative analysis of global gene expression profiles across this developmental series revealed increases in the expression of genes associated with the light-dependent reactions of photosynthesis and reductions in markers of cell proliferation (Figure 3B). Similar to the analysis of unique transcripts, the majority of statistically significant changes in transcript abundance (2,233 of transcripts or 14% of the total annotated) occurred between base and mid sections of the leaf, compared with only 414 transcripts (3% of total) being differentially expressed between mid and tip (Table S2). During an analogous leaf development series in maize more genes were found to be unique to each stage [12]. This is likely due both to the maize genome sequence allowing detection of lower abundance transcripts than permitted by *de novo* assembly as well as ontogenetic differences between the species.

We classified the differentially expressed genes in *C. gynandra* along this development gradient into profiles that showed statistically significant ascending or descending behaviours (Figure 3C). 1,891 and 8,628 genes showed ascending and descending profiles respectively from the base of 3 mm leaves to mature leaves in *C. gynandra* (Figure 3C). In the descending profiles as leaves matured Gene Ontology (GO) terms associated with leaf development, leaf morphogenesis, abaxial-adaxial fate, plasmodesmata, histone acetyl transferase activity and DNA endoreduplication were significantly over-represented (Table S3). These data are consistent with a basipetal source to sink transition as has been observed in C_4_ *Amaranthus hypochondriacus* [29]. We also detected increased abundance of transcripts encoding key enzymes of sucrose biosynthesis and starch degradation from base to tip of 3 mm leaves from *C. gynandra* (Figure S2), further supporting a transition from sink at the leaf base to source at the tip. To gain insight into the extent to which patterns of gene expression are conserved between developing leaves of the C_4_ monocotyledon maize and the C_4_ dicotyledon *C. gynandra* we applied the same profile classification criteria to the maize expression data [12] (Figure 3C). In contrast to *C. gynandra* where approximately 4.5 fold more genes were down-regulated compared with up-regulated as leaves matured, in maize roughly equal numbers of genes increased or decreased along the developmental gradient (Figure 3C). This difference in the dynamics of gene expression in part likely reflects the pronounced developmental differences that discriminate monocots and dicots.

### Differential transcriptome analysis between *C. gynandra* and maize reveals the extent of conservation in leaf development

To define the extent to which these gene expression patterns were conserved between maize and *C. gynandra* we used our orthology assignment method to construct an orthology map linking our *C. gynandra* transcriptome to reference genes in maize. This analysis is therefore designed to discover the extent to which homologous genes occupy common genetic networks underpinning photosynthetic development in these distantly related species. We identified 836 and 2,499 genes whose relative abundance increased and decreased respectively in both species as leaves matured (Figure 3D). These upregulated and downregulated genes encompassed 124 and 121 significantly over-represented GO terms respectively (Table S4). Groups of genes that showed similar patterns in both species included those important for the chloroplast, photosynthesis, response to reactive oxygen species, plasmodesmata, the nucleus, ribosome, proteasome and DNA and RNA binding (Table S4). Genes annotated as being involved in photosynthesis, cell wall and nitrogen metabolism increased as leaves matured in both species, while genes involved in the cell cycle, histone function, nucleotide and protein metabolism decreased.

In the developmental gradient, 216 transcription factors with identifiable homologues in *A. thaliana* exhibited the same expression behaviours in both maize and *C. gynandra* (Figure 3D). Of these, 37 increased while 179 decreased in abundance. Transcription factors with conserved behaviours between the two species are known to play a role in both photosynthetic and leaf development. For example, in the conserved cohort of ascending genes we find *GLK1*, which is implicated in the expression of photosynthesis genes [20], [21], and four Sigma factors associated with transcription of chloroplast photosynthesis genes (Table S5). In the conserved cohort of descending genes, there were multiple *AP2-EREBPs*, *ARFs*, *GRFs* and *TCPs* (Table S5 & Figure S11) that are known to play a role in auxin-mediated development of veins [30] and regulation of cell-cycle and leaf development [31]. The descending conserved cohort also contains genes important in vein patterning such as *SHR*, *PHV*, *HB6* and *PHB* (Table S5 & S6). The identification of transcription factors that have previously been characterised as playing roles in leaf and photosynthetic maturation strongly implies that these 216 regulators fulfil highly conserved roles in these distantly related species.

### Comparative supervised classification identifies marked differences in leaf maturation

**Figure 4:**
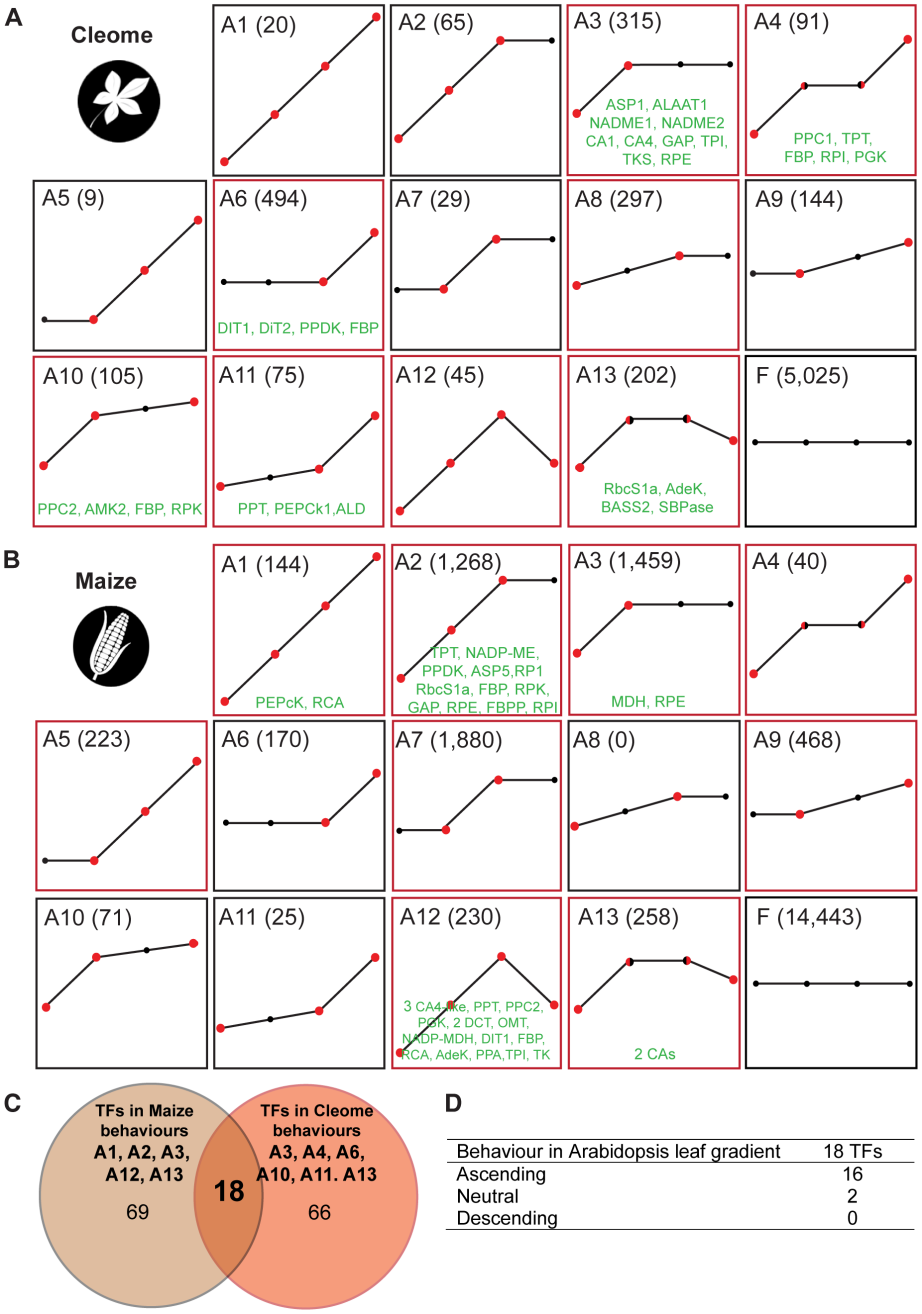
Classification of gene expression in the two C_4_ species *C. gynandra* and maize. As leaves of *C. gynandra* (A) and maize (B) mature, transcripts were classified into twenty-six behaviours, thirteen ascending (A&B) and thirteen descending. Statistically significant differences between neighbouring tissue types are delineated by red circles in ascending filters. The total number of genes within each behaviour is presented in parentheses and behaviours containing photosynthesis-related genes are annotated by red boxes around each plot (eg A3, A4 and A6). Genes of the core C_4_ cycle occupy six and five of the thirteen ascending filters in *C. gynandra* and maize respectively (transcripts in green). (C) Venn diagram representing transcription factors showing the same behaviours as C_4_-related genes in the maize and in *C. gynandra* leaf gradients. (D) Behaviour of homologous genes in C_3_ *A. thaliana*.

We used supervised classification of gene expression to construct profile-based groups containing all differentially expressed genes detected. Using this approach we partitioned all genes into one of twenty-six behaviourally discrete groups, where each group has statistically significant and distinct ascending or descending profiles (Figure 4A&B and Figure S12). Importantly, unlike methods such as k-means clustering, group membership is unbiased by the expression level of individual genes, and is defined by strict statistical criteria. This revealed that, although general behaviour is predominantly conserved, the spatial and temporal separation of genes is markedly different between the two species (Figure 4A&B). Moreover, comparative analysis of each group in *C. gynandra* with each group in maize (and *vice versa*) provided little evidence for a fine-scale unified developmental trajectory between the two species (Figure 4A&B). Therefore, although the global ascending and descending series exhibit marked conservation, and in both cases leaf maturation is occurring, at finer scales of analysis the two species exhibit pronounced differences in patterns of gene expression.

The classification method identified genes that showed a significant change in expression between neighbouring leaf sections (eg base to mid in A3 of *C. gynandra*), but also a significant increase between non-adjacent sections (eg base to tip in A8 of *C. gynandra*). In both species, groups containing the largest number of genes showed either an early or late alteration in transcript abundance (clusters A3&6 and D3&D6, Figure 4A&B and Figure S12), indicating that at the level of gene expression the greatest differences observed were between the base and mid of the developing leaves, and between the tip of developing leaves and mature leaves. Combined with the GO term analysis these data indicate that between the base and middle of developing leaves of *C. gynandra* there were considerable changes in both the number and type of genes expressed, whereas the tip of developing leaves and fully expanded leaves differ with respect to a large number of genes with similar functions. The data are consistent with the ontogenetic framework associated with maturation of C_4_ photosynthesis in leaves of *C. gynandra* (Figure 1). For example, the majority of known regulators of vein production in *A. thaliana* were present in descending clusters as the leaves matured (Figure S13). In addition to increased venation, BS and M size increased from base to tip of *C. gynandra* leaves (Figure 1D). Consistent with this, we detected 186 genes involved in cell expansion that were differentially expressed within the leaf gradient, of which 65 and 121 showed increased and decreased abundance respectively (Table S7). The clustering of genes implicated in chloroplast proliferation was consistent with this process occurring prior to the onset of full photosynthetic capacity, with the majority of transcripts annotated as being involved in chloroplast division decreasing as *C. gynandra* leaves matured but the rates of decline differed (Figure S13). For example, it was notable that *MIND*, *PARC6* and *CLMP1* transcripts declined faster and reached low steady state levels more quickly than the other genes associated with chloroplast division (Figure S13).

### Homologous regulators of C_4_ photosynthesis in M and BS cells of independent C_4_ lineages

In the above analysis photosynthesis genes and genes associated with the C_4_ cycle were not distributed evenly across all expression profiles (Figure 4A). Instead, photosynthesis-related genes populated eight and nine of the ascending clusters in *C. gynandra* and maize respectively (Figure 4A&B and Table S8). Interestingly, genes that encode the canonical C_4_ cycle were only found in profile groups containing photosynthesis-related genes. In maize C_4_ cycle genes were found in five of the nine photosynthesis profiles (Figure 4B) while in *C. gynandra*, C_4_ genes were found in six of the eight photosynthesis profiles (Figure 4A and Table S9). It therefore appears that in both species, genes that comprise the known C_4_ biochemical pathway are co-ordinately regulated with photosynthesis related genes.

To determine if homologous *trans*-factors underlie C_4_ photosynthetic development in *C. gynandra* and maize we compared transcription factors that populated behaviours containing C_4_ genes in both species (Figure 4C and Table S10). This identified a set of 18 transcription factors that are positively co-ordinately expressed with C_4_ genes in both *C. gynandra* and maize (Table S11). Monte Carlo simulation indicated that it is extremely unlikely (p = 0.005) for this number of homologous transcription factors to be present in two equivalent populations of genes by chance. Overall, these data are strongly indicative of a global regulatory role for these transcription factors in promoting and maintaining C_4_ photosynthesis in both species. Interrogating publicly available microarray data obtained from a leaf maturation series of the C_3_ plant *A. thaliana* [23], we found that sixteen of these eighteen transcription factors exhibited analogous expression behaviour in C_3_ leaves (Figure 4D). We therefore propose that these sixteen transcription factors have been recruited from a role in leaf maturation in C_3_ plants into regulating genes of the C_4_ cycle in C_4_ species. This finding also strongly implies that this cohort of sixteen regulators plays a conserved role in leaf maturation in many angiosperms.

**Figure 5:**
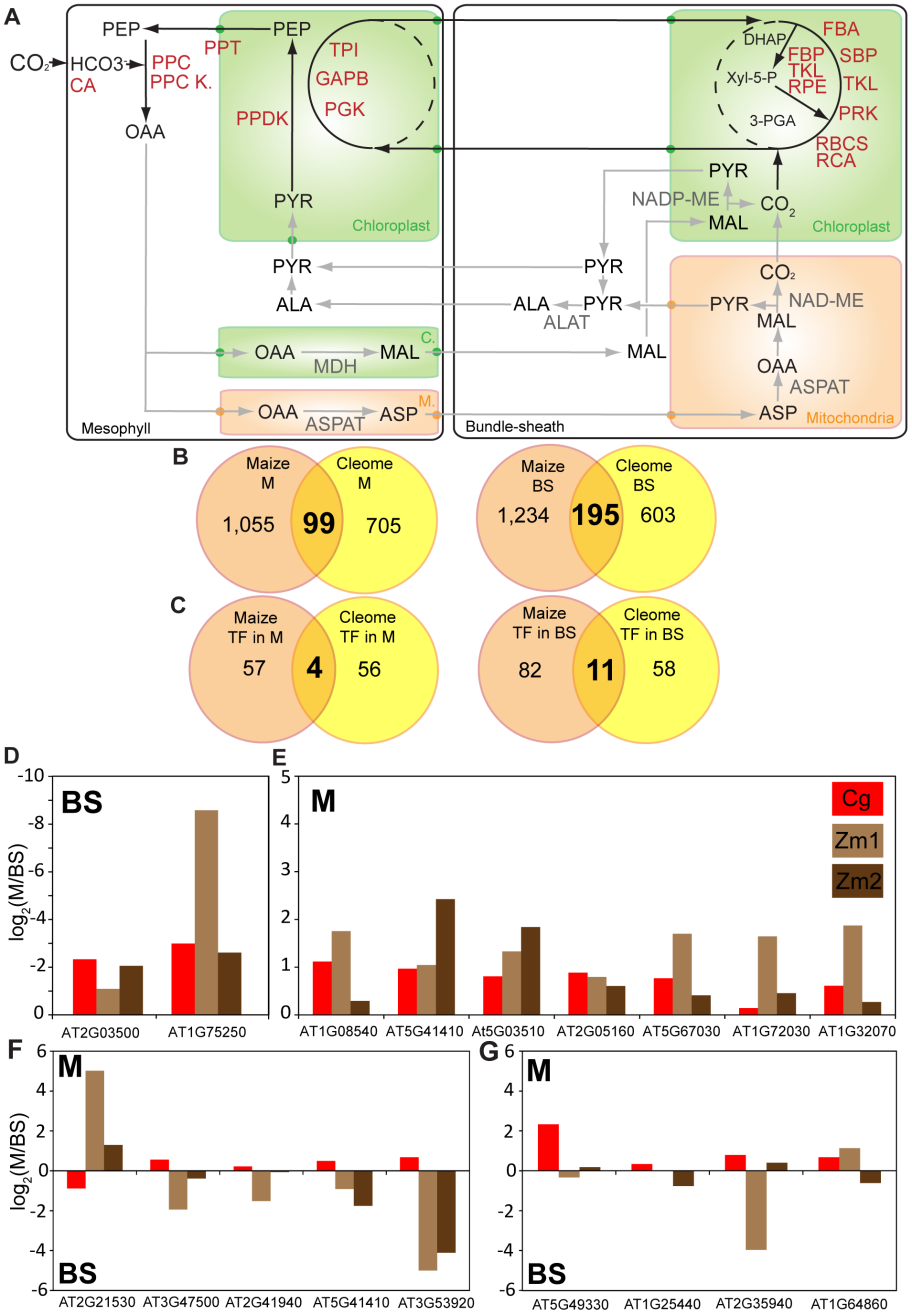
Convergence of mesophyll and bundle sheath transcriptomes in *C. gynandra* and maize. (A) Schematic showing M or BS accumulation of transcripts involved in the C_4_ cycle. Shared parts of the pathway are annotated in red, while differences between the species are shown in grey. CA, carbonic anhydrase; PPC, phospho*enol*pyruvate carboxylase; PEPC Kin, phospho*enol*pyruvate carboxylase kinase, ASPAT, aspartate aminotransferase; ALAAT, alanine aminotransferase; PPDK, pyruvate-orthophosphate dikinase; TPI, triose phosphate isomerase; PGK, phosphoglycerate kinase; FBA, fructose-bisphosphate aldolase; SBP, sedoheptulose-bisphosphatase; TKL, transketolase; PRK, phosphoribulokinase; RbcS, RubisCO small subunit; RCA, RubisCO activase; FBP, fructose 1,6-bisphosphate phosphatase; RPE, D-ribulose-5-phosphate-3-epimerase; NAD-ME, NAD-dependent malic enzyme, MDH malate dehydrogenase. (B) Venn diagrams representing transcripts expressed in M (left panel) and BS (right panel) of *C. gynandra* and maize. Cell-specific maize data represents the overlap between two independent experiments [12], [56]. (C) Venn diagrams of transcription factors expressed in M or BS in maize and *C. gynandra*. (D–G) Expression in M and BS cells of the 18 homologous transcription factors showing co-ordinated induction with C_4_ photosynthesis genes during leaf maturation of both maize and *C. gynandra*. Abbreviations: Cg data from *C. gynandra* (this study), while Zm1 data are from Li *et al* (2010) [12] and Chang et al (2012) [35] respectively.

Once leaf maturation has taken place the C_4_ pathway requires compartmentation of gene expression between M and BS cells to be maintained. We therefore used laser microdissection of M and BS cells from *C. gynandra* followed by Illumina sequencing to investigate the extent to which the transcriptomes of these cell types from *C. gynandra* and maize are convergent. In *C. gynandra*, we detected 13,615 genes (Table S12), of which 338 were significantly more abundant in BS cells while 372 were more abundant in M cells. Despite *C. gynandra* and maize using different C_4_ biochemistries with major flux in maize being maintained by chloroplastic NADP-ME whilst *C. gynandra* using mitochondrial NAD-ME we detected convergence in the expression of many C_4_ cycle genes. For example, genes that encode known components of the C_4_ cycle showed the expected cell specificity (Figure 5A). Most exceptions in convergence relate to the known differences in biochemistry used by the species, for example *C. gynandra* and maize using NAD-ME and NADP-ME respectively. However, we noted that transcripts encoding PEPCK increased dramatically in maize, but decreased in *C. gynandra*, and while transcripts encoding chloroplastic malate dehydrogenase were abundant in maize, this was not the case in *C. gynandra*. We also detected a steady increase in abundance of transcripts encoding NADP-ME, although this protein is not considered to allow major flux through the C_4_ pathway in *C. gynandra* [32], [33]. Lastly, we detected transcripts predicted to encode the mitochondrial ASPAT [34] in the BS of *C. gynandra*.

We compared the cell specific transcriptome from *C. gynandra* with two analogous transcriptome studies from maize [12], [35] and Table S13). This identified 99 and 195 genes that accumulated preferentially in M or BS cells respectively of both species (Figure 5B), of which four and eleven were transcription factors (Figure 5C). Furthermore, of the 18 homologous transcription factors in *C. gynandra* and maize that were co-ordinately expressed with C_4_ genes (Figure 4B), the majority were preferentially expressed in one cell type (Figure 5D–G). For example, two and seven were at least twofold more abundant in M or BS cells respectively (Figure 5D & 5E), five were preferentially expressed in opposite cell types in the two species (Figure 5F), and four showed equal expression in both cell types (Figure 5G). Publically available data derived from laser capture microdissection of M and BS cells in C_3_ rice followed by microarrays [36] detected 7,839 genes, of which 1,392 and 295 were differentially expressed in rice BS and M cells respectively. Whilst rice homologues to 50 of the 195 genes that were highly expressed in C_4_ BS cells (Figure 5B) were detected, only 5 of these were preferentially expressed in the BS of rice. For the structural genes preferentially expressed in C_4_ M cells (Figure 5B), 25 homologues were detected in rice but none of these were preferentially expressed in the M. Furthermore, homologues to five of the *trans*-factors that accumulated preferentially in either C_4_ M or BS cells (Figure 5C) were detected in rice leaves, but none were preferentially expressed in either cell type (Table S15). Taken together, these data indicate that parallel evolution of structural genes as well as transcription factors underlies patterns of gene expression associated with leaf maturation and also cell specificity in these distantly related independent C_4_ lineages.

## Discussion

Our analysis of gene expression in the dicotyledon *C. gynandra* and monocotyledon maize provides insight into the molecular processes underlying C_4_ photosynthesis in these distantly related lineages, but also into leaf maturation more generally. Despite the remarkably different physical scale, temporal scale and large phylogenetic distance between these species we demonstrated that 3,335 genes (comprising 44% of all differentially expressed genes, and ∼10% of genes in the genomes) exhibited analogous expression behaviours during leaf development and photosynthetic induction. As expected this included genes associated with the chloroplast and photosynthesis, but we also found gene categories relating to response to reactive oxygen species, plasmodesmata, the nucleus, ribosome, proteasome and DNA and RNA binding behaving in the same manner. This large overlap is indicative of a core conserved genetic network that regulates leaf development and photosynthetic induction. We discovered 216 transcription factors that exhibited analogous expression behaviours within the constraints of the ontogenetic frameworks of both maize and *C. gynandra* developing leaves. This result implies that there is significant conservation in the *trans*-acting factors in these distantly related species associated with leaf maturation and photosynthetic function. Supporting this conclusion we detected *GLK1*, known to regulate the expression of photosynthesis genes [20], [21], four Sigma factors associated with transcription of chloroplast photosynthesis genes and multiple transcription factors known to regulate vein development [30], cell-cycle and leaf development [31] that behaved the same in both species. These findings suggest that although species-specific differences in mRNA abundance are numerous, the expression profiles of a significant number of transcription factors underpinning both morphological and biochemical development are conserved in *C. gynandra* and maize. These patterns of gene expression are also consistent with those reported in *A. thaliana* during leaf development [23], and so these three datasets encapsulate a conserved molecular toolkit associated with leaf maturation. It is possible these broad-scale similarities in the patterns of gene expression between maize, *A. thaliana* and *C. gynandra* is associated with convergent evolution, but the most parsimonious explanation for these distantly-related species possessing similar patterns of gene expression is that these behaviours are derived from the last common ancestor. We therefore infer that these genes encode proteins that are essential to leaf maturation in all angiosperms derived from the last common ancestor of these species that is estimated to date to around 140 million years ago [37].

With respect to C_4_ photosynthesis in particular, and compared with other C_4_ species assessed to date [12], [38], [39], *C. gynandra* develops C_4_ traits over a short ontogenetic framework of just 3 mm. Similar gradients in leaf maturation have recently been reported in both *C. gynandra* and *Cleome angustifolia*, and immunolocalisation showed selective localisation of RuBisCO in chloroplasts of the BS prior to structural differentiation of M and BS cells [24]. This suggests in *C. gynandra*, *trans*-acting factors which are selectively expressed in M or BS cells, and which appear early in the basal section of the leaf, may be candidates for cell specific control of synthesis of some C_4_ enzymes [24], [40]. Equivalent gradients in gene expression are detected along ∼10 cm of maize leaf. In addition to conservation in global patterns of gene expression associated with leaf development we were also able to show that genes important for the C_4_ pathway show similar patterns of expression to genes annotated as photosynthesis-related. While the concept that genes encoding proteins of the C_4_ photosynthetic pathway should be regulated by existing photosynthesis networks is intuitive, this has not been demonstrated previously. Although the relative abundance of mRNAs from photosynthesis genes increased from base to tip of both species, the rate of increase and point at which steady state was reached varied. Despite this variation in accumulation rate, photosynthesis genes were tightly co-regulated in both species, occupying only eight and nine discrete clusters in maize and *C. gynandra* respectively. There was little evidence for a one-to-one relationship between individual clusters indicating that there is significant divergence in timing and spatial arrangement of photosynthetic and metabolic maturation between the monocotyledons and dicotyledons. As C_4_ pathway genes were distributed among different photosynthetic clusters in *C. gynandra* and maize we were able to identify a small set of transcription factors that were co-ordinately expressed with C_4_ photosynthesis genes in both species. Although this list only contained eighteen transcription factors, this is significantly enriched compared to the background rate of transcription factor co-expression for sets of genes of the same size. Furthermore, we found that 16 of these 18 transcription factors exhibited analogous expression behaviour in the C_3_ leaves of *A. thaliana*. This finding strongly implies that this cohort of regulators plays a conserved role in leaf maturation in many angiosperms and has been co-opted to regulate C_4_ pathway genes in both *C. gynandra* and maize. The fact that fourteen of these eighteen transcription factors accumulate preferentially either in M or BS cells of *C. gynandra*, and that nine show exactly the same distribution in maize indicates that they very likely underlie regulation of components required for the C_4_ pathway. We propose that the five transcription factors with preferential but opposite patterns of expression in M and BS cells of maize and *C. gynandra* underpin differences in gene expression associated with their belonging to the distinct NAD-ME and NADP-ME biochemical subtypes. Of the eighteen transcription factors that we detected as showing the same behaviours in *C. gynandra* and maize, only a subset were detected by an independent microarray analysis of maize leaf maturation [11], but of these, the majority increased in abundance as leaves matured, further supporting a role in C_4_ maturation (Table S14).

In addition to similarities in the patterns of gene expression as leaves of *C. gynandra* and maize matured, we also found significant overlap in gene expression of M and BS cells between these two independent C_4_ lineages. In maize 21–25% of all genes expressed in leaves were estimated to be differentially expressed in M and BS cells [13], [24]. As these maize experiments were conducted with very different technologies there are significant differences in their estimates of gene expression, however we found 1,154 and 1,429 genes that were preferentially expressed in the M or BS respectively in both maize datasets. Furthermore, of the maize genes that were consistently differentially expressed in the M and BS, 99 and 195 were homologous to M and BS specific genes in *C. gynandra*. These data indicate that in the two species these cell types show more differences in gene expression than similarities. However, we did detect fourteen directly homologous transcription factors specific to each cell type in both species. These data are consistent either with these transcription factors playing fundamental conserved roles in M and BS cells of all C_3_ as well as C_4_ species, or that they have been recruited into regulate processes relating to C_4_ photosynthesis in independent C_4_ lineages. Previous analysis of transcript abundance in M and BS cells of C_3_ rice [36] provides insight into the extent to which cell specific expression of these genes is an ancestral characteristic, or, whether this cell specialisation has occurred in parallel in both C_4_ lineages. Whilst, fewer genes were detected in the rice microarray study [36], homologues to five of the *trans*-factors preferentially expressed in BS or M cells of both *C. gynandra* and maize were detected. However, none of these were preferentially expressed in the BS or M cells of rice, strongly implying parallel recruitment into specific roles in these cells in independent C_4_ lineages.

In summary, our data indicate that a broad comparative approach of distantly related species can shed light on the molecular signatures of highly complex traits. With respect to C_4_ photosynthesis in particular, we show that not only is it underpinned by the parallel evolution of *cis*-elements [41] and amino acid substitutions [42], but also that expression of homologous transcription factors follow analogous temporal and spatial patterns of expression in independent lineages of C_4_ plants. Additional studies of C_4_ species with structurally similar leaves but differing types of C_4_ biochemistry may well help identify *trans*-factors that act to regulate structural versus biochemical development of C_4_ function. For this to be efficient, the developmental stage at which samples are taken will need to be carefully selected [43]. As key regulators generating the C_4_ phenotype are shared between lineages, this opens up the possibility of using natural variation to identify regulators and therefore to facilitate engineering C_4_ photosynthesis into C_3_ crops [44] to increase their yield.

## Materials and Methods

*C. gynandra* was grown in soil under long-day conditions in a cabinet with light intensity of 150 µmol photons m^−2^ s^−1^ and a temperature of daytime 23°C/20°C. Four hours after dawn, RNA was extracted (Plant RNeasy kit, Qiagen) from at least 100 mg of leaf material from at least three plants for each biological replicate. The amount and quality of RNA was determined using a Bionanalyzer RNA 6000 nanochip (Agilent). The poly(A)^+^ RNA was isolated and sequenced using standard illumina protocols on a HiSeq to generate 3 Gb of 90 bp pair ended reads for each biological replicate. Each gradient condition (base, mid, tip, mature) is a mixture of at least 50 leaves and three replicates for each condition have been sequenced on the same flow cell.

### Histology, quantification of venation pattern

To assess venation leaves were fixed in 70% ethanol at 65°C prior to clearing in 5% (w/v) NaOH [45]. Venation density (vein length per unit area) and complexity (sum of the number of end-points, branching points and vascular elements) were quantified using LIMANI [46]. For cell size analysis, tissue was fixed in glutaraldehyde/paraformaldehyde and embedded in Teknovit 7100 resin. 2 µm thick sections were made and then stained with toluidine blue [45]. For transmission electron microscopy, 50 nm thick sections were cut with a Leica Ultracut UCT, stained with saturated uranyl acetate in 50% ethanol and lead citrate, and viewed in a FEI Philips CM100 operated at 80 kV.

### Assembling, annotation and estimation of transcript abundance

Paired end reads were subject to quality-based trimming using the FASTX toolkit [47] setting the PHRED quality threshold at 20 and discarding reads less than 21 nucleotides in length. Further processing was then performed to remove reads corresponding to poly-A tails and reads containing more than 75% of any single nucleotide. These processed reads were then subject to read error correction using the ALLPATHS-LG [48] and then filtered to remove all redundant read-pairs. Finally reads containing only unique kmers were discarded. This processed read set was then subject to *de novo* assembly using velvet/oases [27], [49] using four different kmer lengths (k = 31, 41, 51, 61) and merged using oases. Redundant transcripts and partial transcripts (for which a longer transcript was present that contained >95% of the nucleotides of the shorter) were discarded using usearch [50]. To estimate transcript abundances the original unprocessed reads were subject to quality-based trimming using the FASTX toolkit [47] setting the PHRED quality threshold at 20 and discarding reads less than 21 nucleotides in length. These trimmed reads were then used to quantify the assembled transcripts using RSEM [51]. Read library sizes and Spearman's ranked correlation coefficients between all samples and replicates (computed using all expressed genes) are provided in Figure S14.

*De novo* assembled transcript sequences with homologues in the genome of *Arabidopsis thaliana* were identified using the conditional orthology assignment method described and validated in the supplemental methods (Text S1). Annotation information including GO terms and MapMan classifications already assigned to *Arabidopsis thaliana* genes were directly allocated to the newly identified homologous in the *de novo* assembly.

All possible pairwise comparisons between replicated samples were performed using DESeq [52]. Prior to differential testing, RNAseq count data were normalised between conditions to account for differences in library size and any lane biases using the median ratios method employed in DESeq. In all cases, differentially expressed genes were identified as those genes with a Benjamini-Hochberg corrected p-value of less than 0.05 [53]. Supervised classification of gene expression profiles was performed using p-values and normalised, replicate-averaged expression estimates derived from DESeq. For all enrichment testing, significant enrichment was identified as gene groups with a Benjamini-Hochberg corrected p-value of less than 0.05 following Wallenius approximation and length normalisation of uncorrected p-values using goseq [54]. The probability that 18 transcription factors would be found in C_4_ behaviours in both species by chance was evaluated by Monte Carlo simulation. For each sample, twenty-nine genes (the number of C_4_ cycle genes) were randomly selected to define sets of expression behaviours. The number of transcription factors that were present in these behaviours in both species was determined. This procedure was repeated one million times to build the reference distribution of transcription factors occurring in the gene lists of both species by chance, and to calculate an empirical p-value.

### Laser capture microdissection

Leaf tissue was harvested 4 hrs after dawn and immediately infiltrated in ethanol∶acetic acid (3∶1). The tissue was processed through a series of dehydration and then replaced by Paraplast Xtra (Sigma). Leaves embedded in wax were sectioned transversely using 8 µm thin sections. Sections were floated in EtOH on MembraneSlide 1.0 PEN (Zeiss) and dried. For laser capture microdissection (LCM), slides were deparaffinised using Histo-clear for 2 min and air dried. LCM was performed using Arcturus XT (Life Technologies) and mesophyll and bundle-sheath were captured using adhesive caps (Life Technologies) following manufacturer instructions. Subsequently RNA was purified using Picopure RNA extraction kit (Life Technologies) and subjected to on-column DNAse treatment (Qiagen) and amplified using Nugen RNA Ovation V2 kit (Nugen) according to the manufacturer's instructions. RNA quality and quantities were checked at every stage using a picoRNA chip on Bioanalyzer 2100 (Agilent). Amplified cDNA libraries were using the Illumina standard protocol and then multiplexed on HiSeq to generate 2 Gb of 100 bp pair ended reads for each library (in triplicate for each cell type).

### Quantification of cell specific transcriptomes in *C. gynandra* and maize

Raw reads for the maize cell specific transcriptomes [12], [35] were downloaded from NCBI SRA. All read datasets (including those from *C. gynandra*) were subject to the same quality based trimming prior to quantification using RSEM as described above. All possible pairwise comparisons between replicated samples were performed using DESeq and differentially expressed genes were identified as those genes with p-value of less than 0.05. Only genes which exhibited the same cell type specificity in both maize datasets were considered to be differentially expressed in maize.

### Real-time quantitative PCR

First-strand cDNA synthesis from 0.5 µg RNA was performed using Superscript II (Invitrogen) prior to quantitative real-Time PCR using SYBRgreen Jumpstart (Sigma) in a rotor-gene-Q system (Qiagen). Gene specific primers were designed according to contigs assembled during the analysis. The relative expression was normalised based an external alien qRT-PCR RNA spike (Agilent). For each gene assessed three technical and three biological replicates were carried out.

### Immunoblots

After separation by SDS-PAGE, proteins were transferred to nitrocellulose membranes according to standard procedures. Proteins were detected with polyclonal antibodies against rice CA (1∶5000), maize PPC (1∶5000), the α-subunit of NAD-ME (1∶5000) and PPDK (1∶10,000) as in [32] and were a gift from Richard Leegood (University of Sheffield. UK). Subsequently, the membranes were labelled with anti-rabbit secondary antibody (1∶10,000) coupled to HRP (Sigma) and visualised by chemoluminescence using Western lightning Plus-ECL (Perkin-Elmer). For each protein assessed immunoblots were carried out on duplicates.

### Accession numbers

RNAseq data produced in this study have been submitted to the NCBI/SRA database under accession number SRA066236.

## Supporting Information

Figure S1 BS cells contain slightly bigger chloroplasts (A–D&I) than mesophyll cells (E–H) along the gradient. Granal stacking is similar in BS and M chloroplasts. Plasmodesmata can be observed in the base of 3 mm leaves (J–K). Data are derived from at least three sections from each species. Scale bars = 500 nm.
(TIF)

Figure S2 Expression along *C. gynandra* leaf gradient for (A) sucrose synthesis and (B) starch synthesis enzymes indicating a sink-to-source transition. Data is shown as normalized read counts (see Materials and Methods) for Base (B), Mid (M), Tip (T), and Mature (Mat.) leaves.
(TIF)

Figure S3 Flow diagram of the conditional orthology assignment method. (A) The method begins by performing all versus all BLAST searches of the assembled transcripts against a reference proteome. (B) The reciprocating hits (indicated by blue lines) are selected for self-training. (C) The reciprocating hits are binned according to assembled transcript length and a quadratic model is fit to the e-value and length data. D) Non-reciprocating hits which fall above the curve are accepted as putative homologues, non-reciprocating hits which fall below the curve are rejected.
(TIF)

Figure S4 Quantitative differences in generic metrics between test assemblies. (A) Maximum observed transcript length. (B) Mean transcript length. (C) Median transcript length. (D) N50. (E) Number of transcripts longer than 1000 bp. (F) Number of transcripts longer than the read length (100 bp). For each panel the number indicates the k-mer length used in the assembly. nrec means that the assembly was performed on **n**on-**r**edundant **e**rror **c**orrected reads. qcnrec means that the assembly was performed on **q**uality **c**lipped **n**on-**r**edundant **e**rror-**c**orrected reads. Merged indicates a merged sample which contains the assemblies produced from all k-mer sizes.
(TIF)

Figure S5 Qualitative differences in transcriptome content between test assemblies. (A) The mean number of assembled transcripts which hit each reference transcript. (B) The proportion of the reference transcriptome which have hits with e-values better than 1×10^−5^ in the assembled transcriptome. (C) The mean number of reference transcripts which hit each assembled transcript. (D) The proportion of assembled transcripts which have hits with e-values better than 1×10^−5^ in the reference transcriptome. For abbreviations see legend to Figure S3.
(TIF)

Figure S6 The effect of read processing and k-mer size selection on detection of reference transcripts. In each case the Venn diagram represents the overlap in detected reference transcripts between each of the assemblies. (A) Assemblies made from raw unprocessed sequence reads. (B) Assemblies made from non-redundant, error corrected sequence reads. (C) Assemblies made from non-redundant, error corrected and quality-clipped sequence reads. The k-mer size is indicated next to each oval and the total number of transcripts contained in the entire set is indicated below.
(TIF)

Figure S7 The effect of read processing and k-mer size selection on the accuracy of transcript abundance estimates. (A) Correlation in quantification derived from reciprocal best BLAST (RBB) hits in the assemblies and reference. (B) As in (A) but summed over all transcript isoforms per reference gene locus. For abbreviations see legend to Figure S3. (C) Scatter plot comparing log transformed read counts obtained from RSEM quantification of the de novo assembly and the reference transcriptome (example shown is the merged non-redundant error corrected assembly). Correlation in quantification derived from reciprocal best BLAST hits in the assemblies and reference. (D) as in (C) but integrated over all transcript isoforms per reference locus. The thin dashed red line indicates the line of equivalent expression. The thick dashed red lines indicate the 25% intervals.
(TIF)

Figure S8 Example of conditional orthology assignment data fitting. (A) Grey dots indicate 1st percentile e-value of reciprocal best BLAST hits. Black line indicates the quadratic polynomial curve fit to the data. This line is used to identify putative homologues. Sequences of a given length that have e-values above the line are considered putative homologues. Those below the line are not. (B) The Spearman correlation in transcript abundances between the reference guided estimation and estimates generated using different transcript orthology assignment methods on the same *de novo* assembled transcriptome. “RBB only” means that only the reciprocal best BLAST transcripts were selected. E-value cut-offs (e.g. 1e-5) indicate the fixed value at which sequences were determined to be homologues. OrthoMCL indicates that OrthoMCL was used to cluster and identify orthologous transcript groups. Finally, the black bar indicates the effect of varying the percentile cut-off on the abundance estimate accuracy of the conditional orthology assignment method.
(TIF)

Figure S9 The effect of conditional orthology assignment on gene expression estimates. (A) Correlation in quantification derived from conditional assigned transcripts. (B) As in (A) but summed over all transcript isoforms per reference locus. For abbreviations see legend to Figure S4. (C) Scatter plot log transformed read counts obtained from RSEM quantification of the conditional assigned *de novo* assembly and the reference transcriptome (example shown is the merged non-redundant error corrected sample). (D) As in (C) but summed over all transcript isoforms per reference locus. The thin dashed red line indicates the line of equivalent expression. The thick dashed red lines indicate the 25% intervals.
(TIF)

Figure S10 The effect of using an intermediary reference proteome to assign transcripts and compare expression data. (A) The effect of percentile cut-off on the homologue detection accuracy (F_1_ score) of the conditional assignment method. (B) Comparison between log transformed read counts in assembled and reference transcriptome using the *Arabidopsis thaliana* transcriptome as an assignment intermediary. The thin dashed red line indicates the line of equivalent expression. The thick dashed red lines indicate the 25% intervals.
(TIF)

Figure S11 Behaviour of all transcription factor families identified within the *C. gynandra* leaf gradient. Transcription factors were classified by family (A) and the proportion of the genes ascending (red) or descending (blue) were assessed for each of the 70 families detected. A schematic leaf (B) shows specific TF families with related functions in function of their expression profile.
(TIF)

Figure S12 Classification of transcripts into thirteen descending behaviours as leaves of C. *gynandra* (A) and maize mature (B). Statistically significant differences between neighbouring tissue types are delineated by blue circles in descending filters, non-significant differences are indicated by black circles. The total number of genes that exhibit each behaviour is presented in parentheses.
(TIF)

Figure S13 Behaviour of genes involved in chloroplast division, positioning and venation in the base, middle and tip of 3 mm leaves as well as mature leaves of *C. gynandra*. Genes annotated as being important for venation were found in two ascending and eight descending filters (A), genes for chloroplast positioning in two descending and two ascending filters (B), and those annotated as being involved in plastid division in seven descending clusters (C). The total number of genes in each filter is annotated in parentheses, and specific genes in each class depicted in green text.
(TIF)

Figure S14 Spearman ranked correlation coefficients for pairwise sample comparisons of global mRNA abundance estimates. Correlation shown as a heatmap (strongest correlation in red, weakest correlation black) with numerical provided below. Triplicate sequencing replicates for each of the 4 tissue sections are shown.
(TIF)

Table S1 List of genes detected along *C. gynandra* leaf gradient.
(XLSX)

Table S2 List of differentially expressed genes in *C. gynandra* leaf gradient and pairwise comparisons (base versus middle, middle versus tip, base versus tip, and tip versus mature).
(XLSX)

Table S3 GO terms that are different between specific tissues within the *C. gynandra* leaf gradient.
(XLSX)

Table S4 GO terms that are different between *C. gynandra* and maize.
(XLSX)

Table S5 Transcription factors present in ascending or descending behaviours in both *C. gynandra* and maize.
(XLSX)

Table S6 Genes showing the same behaviours in both *C. gynandra* and maize.
(XLSX)

Table S7 Genes associated with cell wall expansion
(XLSX)

Table S8 Expression profiles of photosynthesis-related genes in the *C. gynandra* leaf gradient.
(XLSX)

Table S9 Expression profiles of C_4_ pathway genes in *C. gynandra* leaf gradient.
(XLSX)

Table S10 Transcription factors showing the same behaviours in both *C. gynandra* and maize.
(XLSX)

Table S11 Transcription factors in common between *C. gynand*ra and maize after comparative analysis of gene expression in leaf gradients. *Arabidopsis thaliana* accession numbers are listed more than once if multiple paralogues/homeologues exist in the maize genome. Genes were classified in potential positive or negative regulators based on their presence in the C_4_-related clusters.
(XLSX)

Table S12 Genes detected in BS and M cells of *C. gynandra* harvested by LCM.
(XLSX)

Table S13 List of genes overlapping between *C. gynandra* BS and M fractions and *Z. mays* BS and M fractions (data from Li *et al.*, [12], Chang *et al.* [35]).
(XLSX)

Table S14 Homologous transcription factors expressed in C_4_-genes clusters in both *Cleome gynandra* and maize. Data from Pick *et al.* [11].
(XLSX)

Table S15 Expression of transcription factors in rice BS or M cells that are homologous to those defined in maize and *C. gynandra* (Fig. 5C). Data from Jiao *et al.* [36].
(XLSX)

Text S1 Supplementary methods.
(DOCX)
