# Correction: Deep Evolutionary Comparison of Gene Expression Identifies Parallel Recruitment of *Trans*-Factors in Two Independent Origins of C_4_ Photosynthesis

**DOI:** 10.1371/journal.pgen.1006087

**Published:** 2016-05-24

**Authors:** Sylvain Aubry, Steven Kelly, Britta M. C. Kümpers, Richard D. Smith-Unna, Julian M. Hibberd

There is an error in the first sentence of the Materials and Methods. The correct sentence is: *C*. *gynandra* was grown in soil under long-day conditions in a cabinet with light intensity of 350 μmol photons m^-2^ s^-1^ and a temperature of daytime 23°C/20°C.
