# Supplementary figures and images for: Deep Evolutionary Comparison of Gene Expression Identifies Parallel Recruitment of Trans-Factors in Two Independent Origins of C4 Photosynthesis
Source: PLoS Genet. 2014 Jun 5;10(6):e1004365. doi: 10.1371/journal.pgen.1004365 (PMC4046924; doi:10.1371/journal.pgen.1004365)

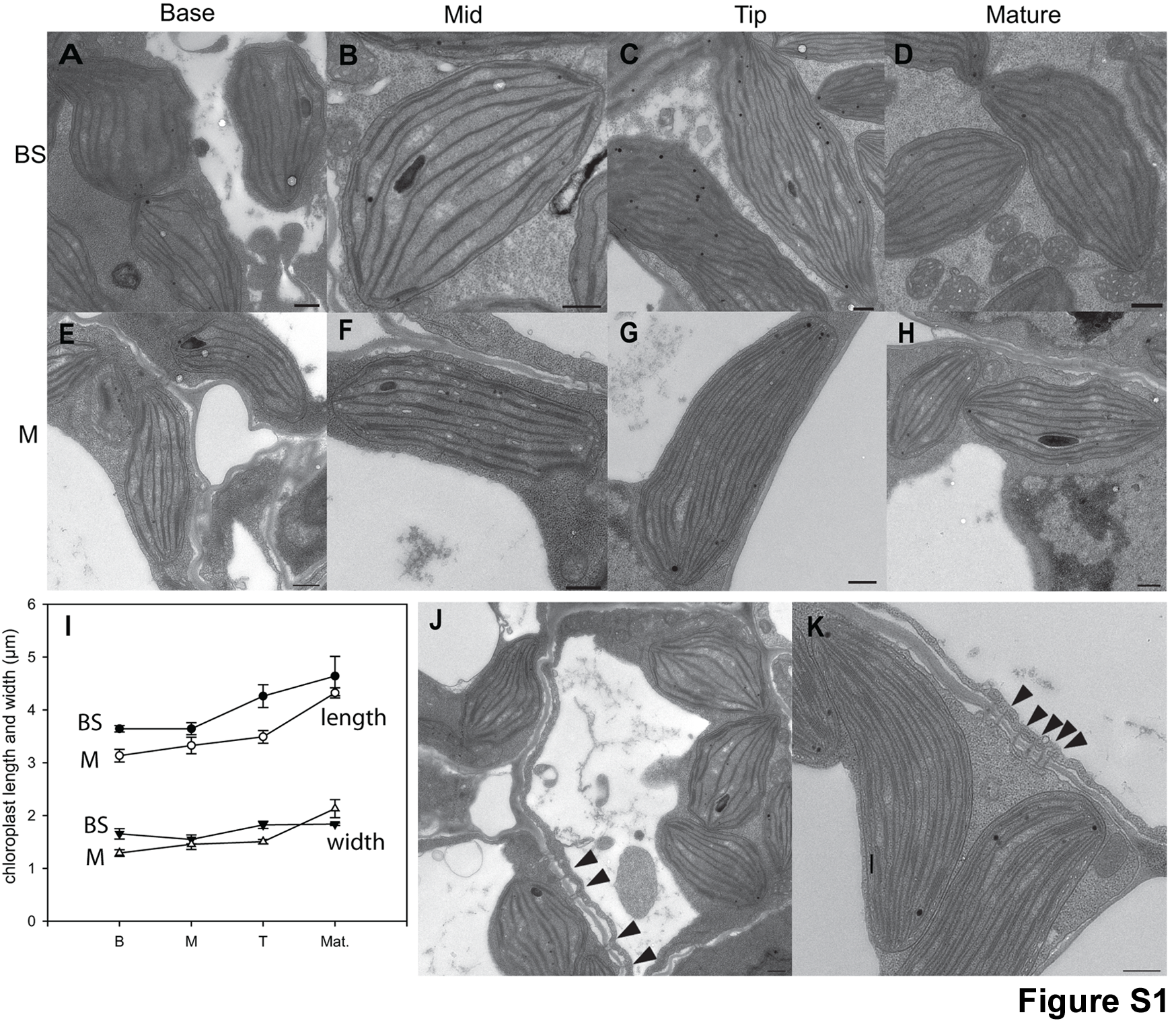

Supplement: Figure S1 — BS cells contain slightly bigger chloroplasts (A–D&I) than mesophyll cells (E–H) along the gradient. Granal stacking is similar in BS and M chloroplasts. Plasmodesmata can be observed in the base of 3 mm leaves (J–K). Data are derived from at least three sections from each species. Scale bars = 500 nm. (TIF) [file pgen.1004365.s001.tif]

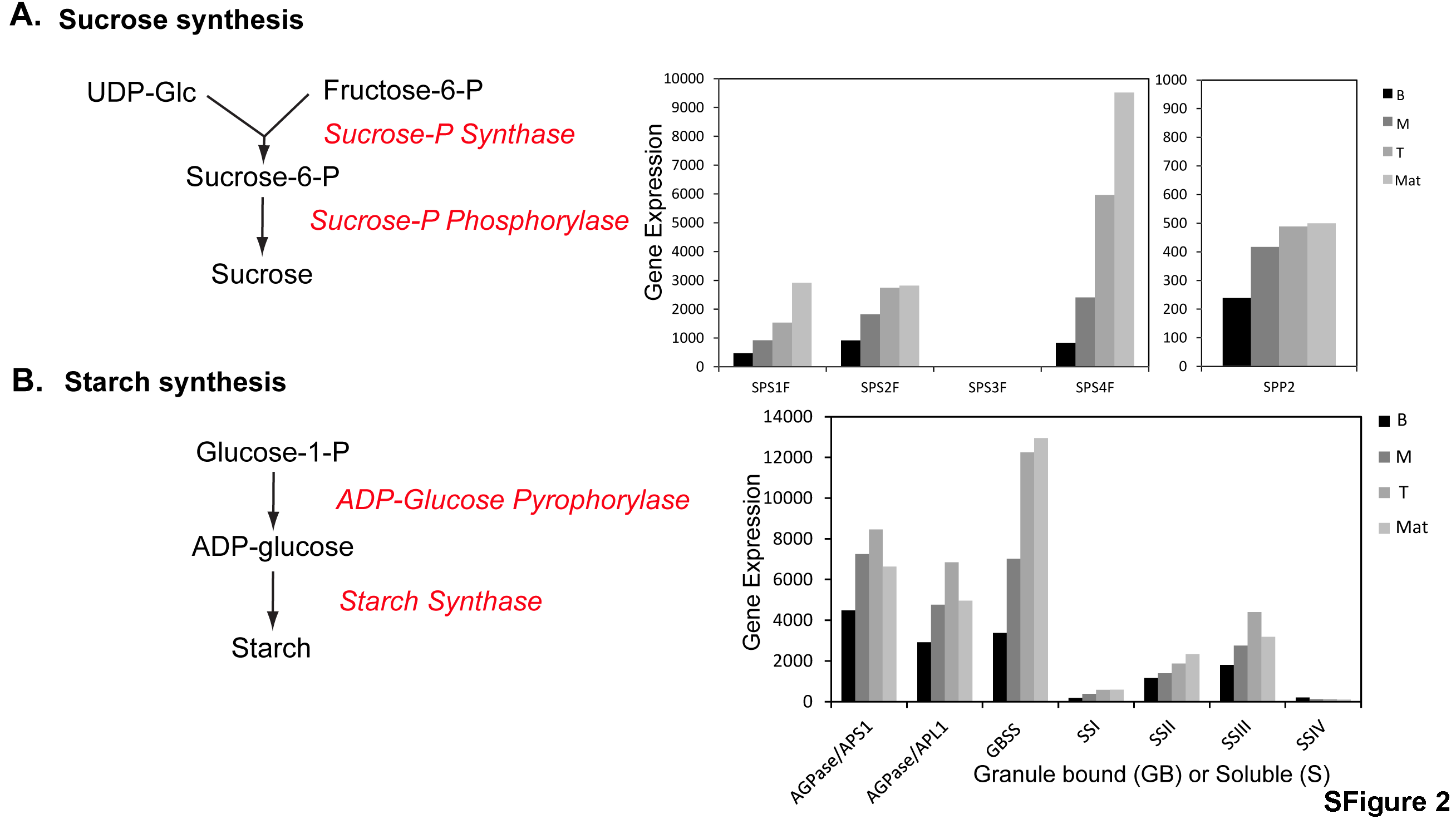

Supplement: Figure S2 — Expression along C. gynandra leaf gradient for (A) sucrose synthesis and (B) starch synthesis enzymes indicating a sink-to-source transition. Data is shown as normalized read counts (see Materials and Methods) for Base (B), Mid (M), Tip (T), and Mature (Mat.) leaves. (TIF) [file pgen.1004365.s002.tif]

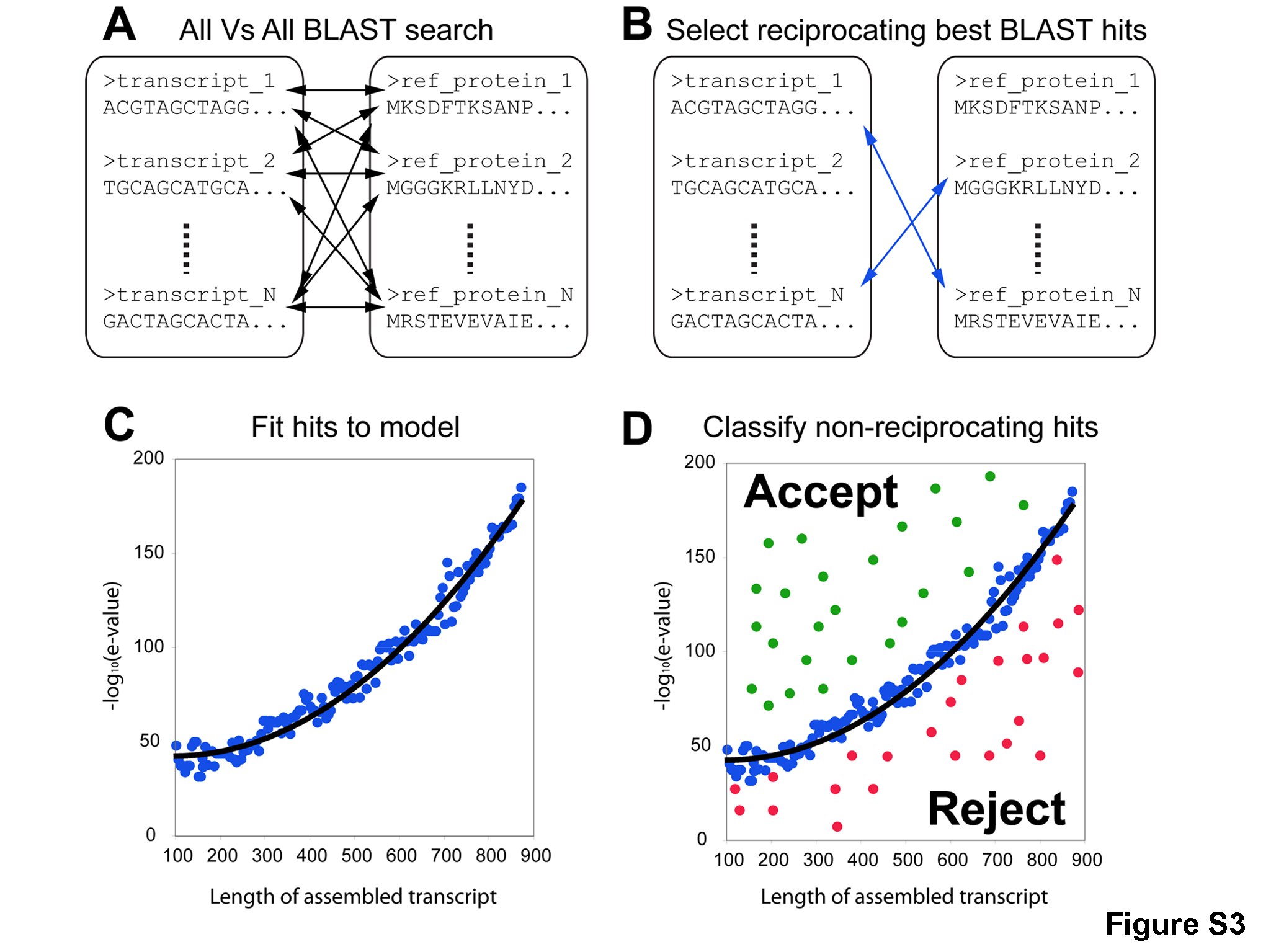

Supplement: Figure S3 — Flow diagram of the conditional orthology assignment method. (A) The method begins by performing all versus all BLAST searches of the assembled transcripts against a reference proteome. (B) The reciprocating hits (indicated by blue lines) are selected for self-training. (C) The reciprocating hits are binned according to assembled transcript length and a quadratic model is fit to the e-value and length data. D) Non-reciprocating hits which fall above the curve are accepted as putative homologues, non-reciprocating hits which fall below the curve are rejected. (TIF) [file pgen.1004365.s003.tif]

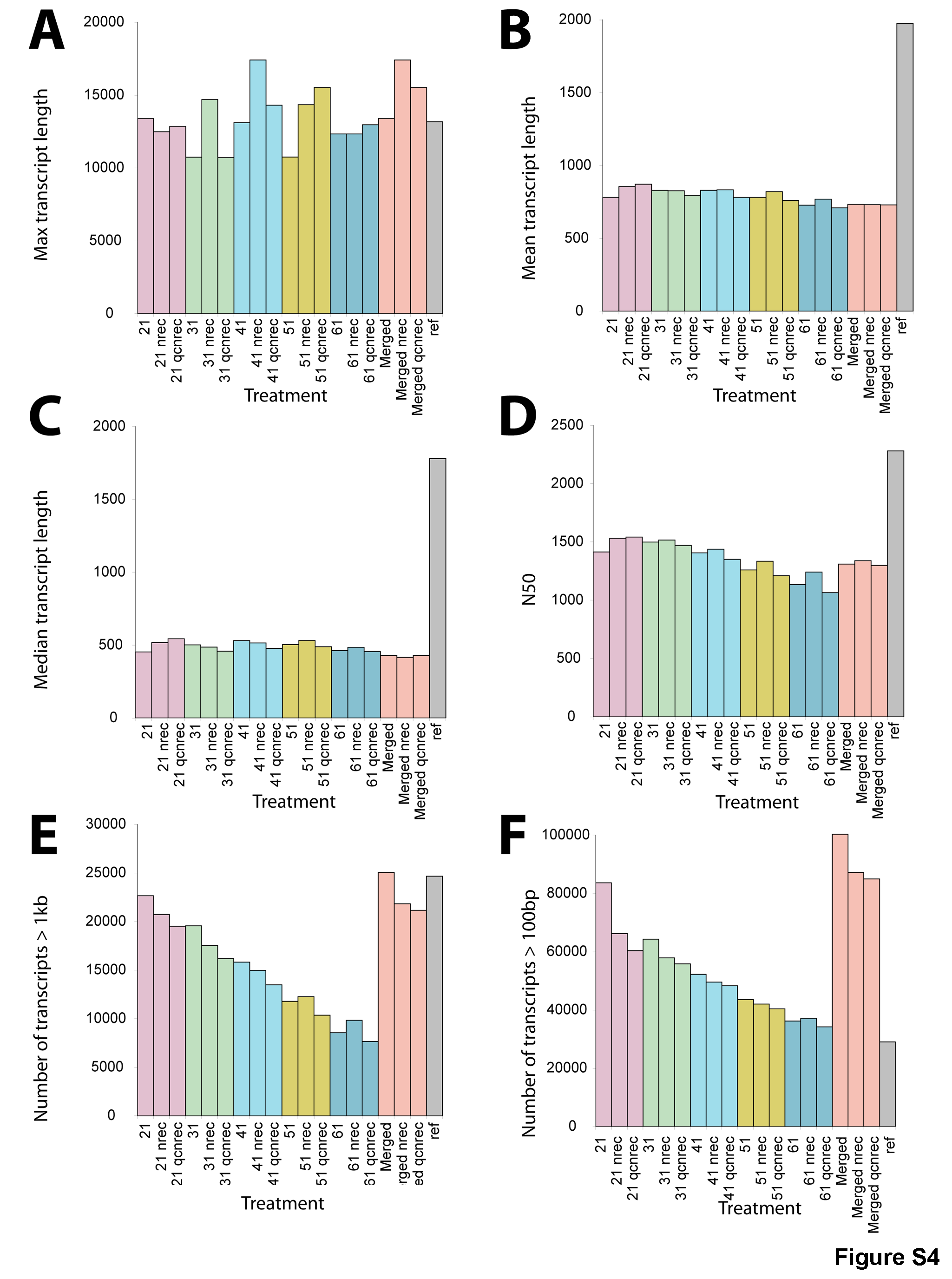

Supplement: Figure S4 — Quantitative differences in generic metrics between test assemblies. (A) Maximum observed transcript length. (B) Mean transcript length. (C) Median transcript length. (D) N50. (E) Number of transcripts longer than 1000 bp. (F) Number of transcripts longer than the read length (100 bp). For each panel the number indicates the k-mer length used in the assembly. nrec means that the assembly was performed on non-redundant error corrected reads. qcnrec means that the assembly was performed on quality clipped non-redundant error-corrected reads. Merged indicates a merged sample which contains the assemblies produced from all k-mer sizes. (TIF) [file pgen.1004365.s004.tif]

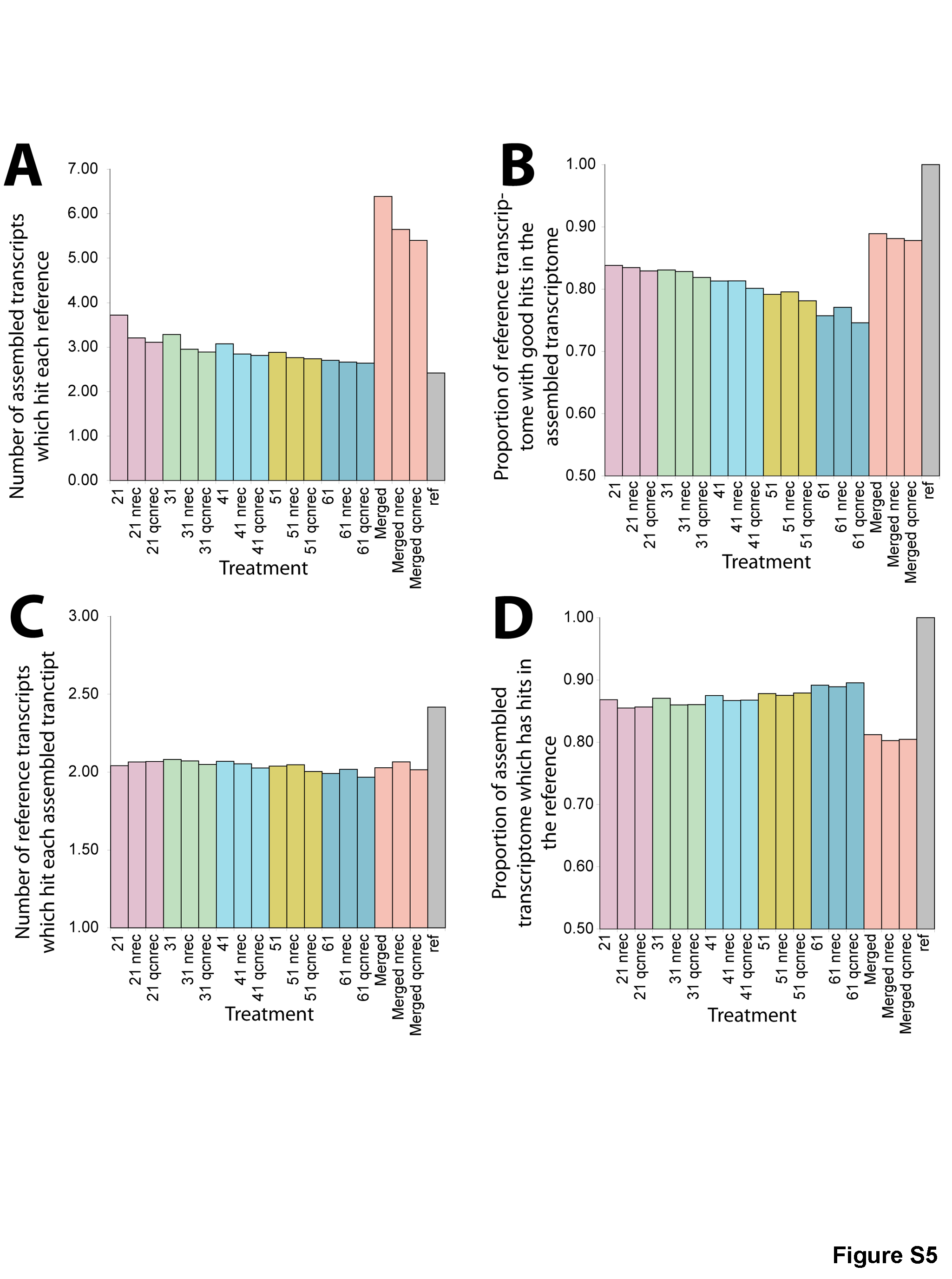

Supplement: Figure S5 — Qualitative differences in transcriptome content between test assemblies. (A) The mean number of assembled transcripts which hit each reference transcript. (B) The proportion of the reference transcriptome which have hits with e-values better than 1×10^−5^ in the assembled transcriptome. (C) The mean number of reference transcripts which hit each assembled transcript. (D) The proportion of assembled transcripts which have hits with e-values better than 1×10^−5^ in the reference transcriptome. For abbreviations see legend to Figure S3. (TIF) [file pgen.1004365.s005.tif]

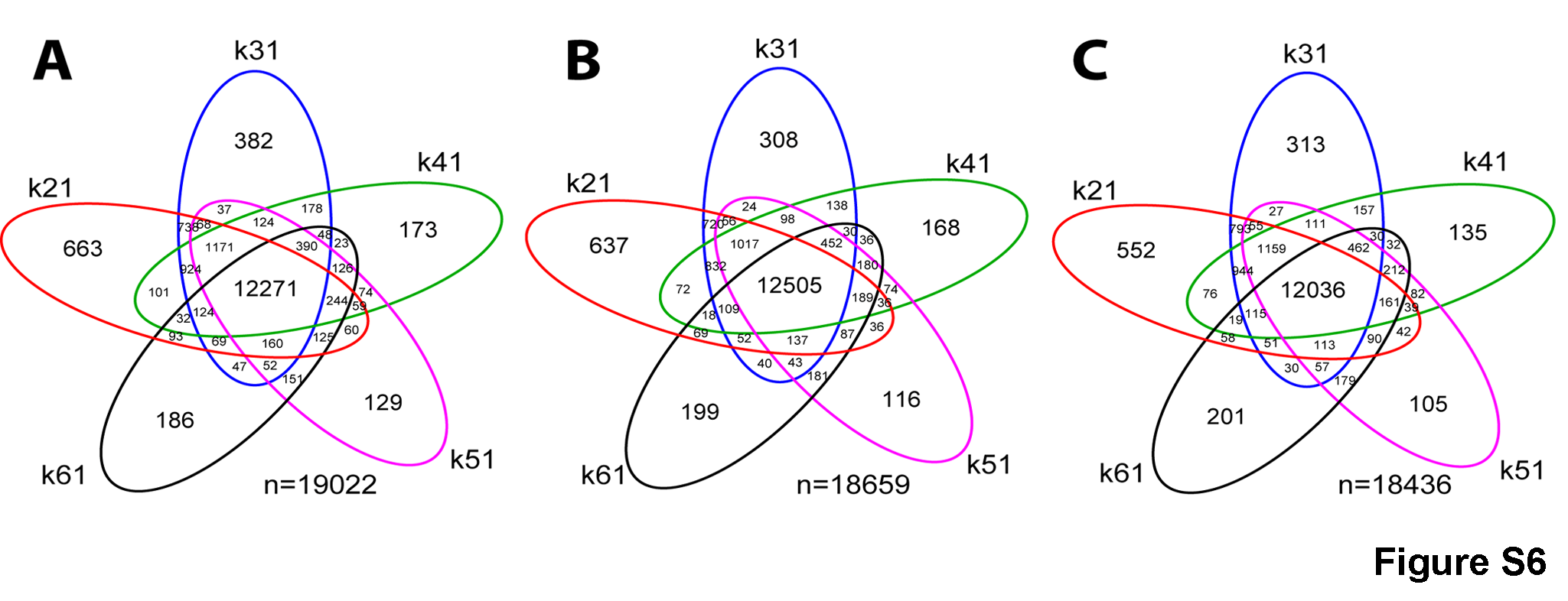

Supplement: Figure S6 — The effect of read processing and k-mer size selection on detection of reference transcripts. In each case the Venn diagram represents the overlap in detected reference transcripts between each of the assemblies. (A) Assemblies made from raw unprocessed sequence reads. (B) Assemblies made from non-redundant, error corrected sequence reads. (C) Assemblies made from non-redundant, error corrected and quality-clipped sequence reads. The k-mer size is indicated next to each oval and the total number of transcripts contained in the entire set is indicated below. (TIF) [file pgen.1004365.s006.tif]

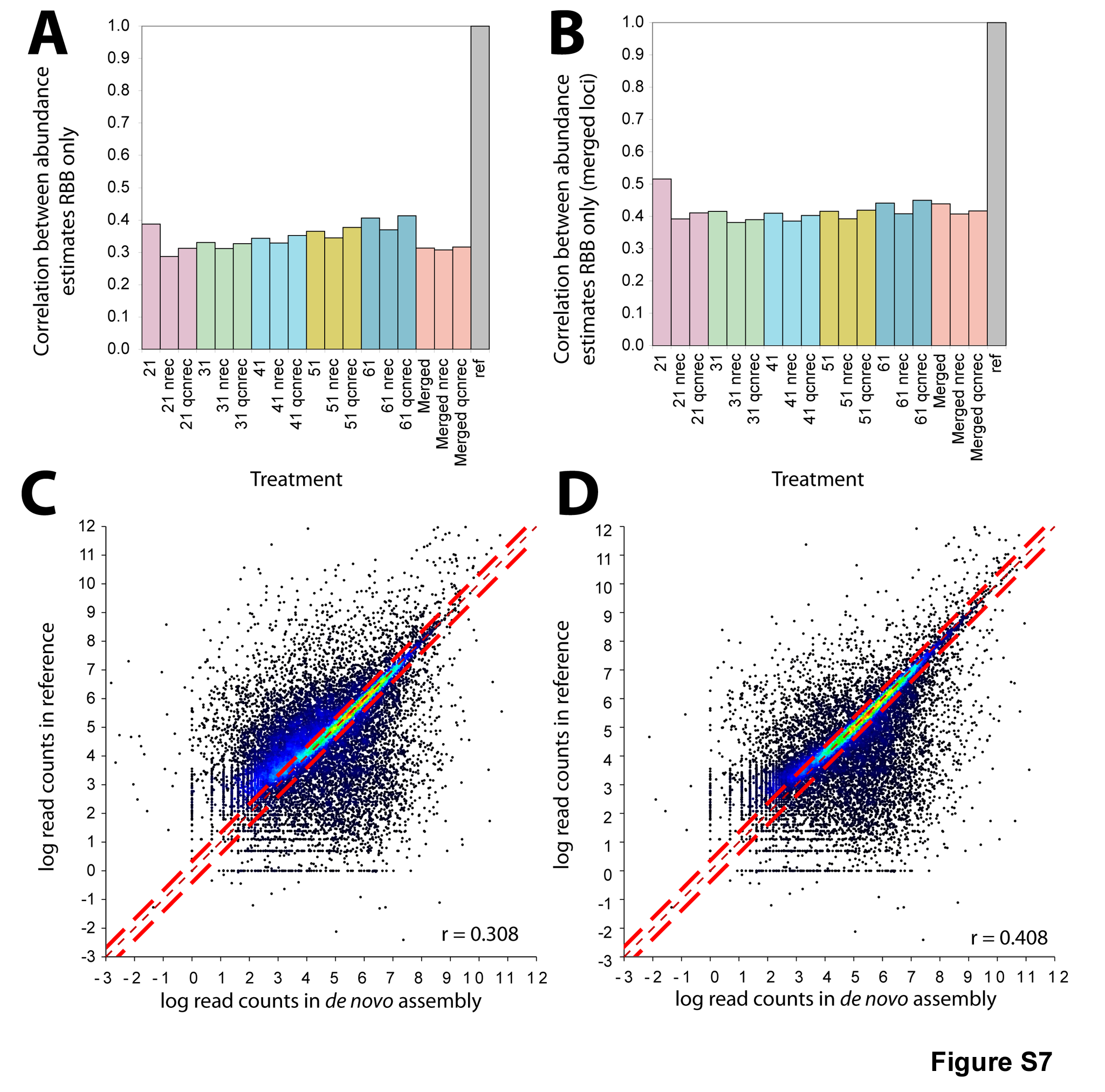

Supplement: Figure S7 — The effect of read processing and k-mer size selection on the accuracy of transcript abundance estimates. (A) Correlation in quantification derived from reciprocal best BLAST (RBB) hits in the assemblies and reference. (B) As in (A) but summed over all transcript isoforms per reference gene locus. For abbreviations see legend to Figure S3. (C) Scatter plot comparing log transformed read counts obtained from RSEM quantification of the de novo assembly and the reference transcriptome (example shown is the merged non-redundant error corrected assembly). Correlation in quantification derived from reciprocal best BLAST hits in the assemblies and reference. (D) as in (C) but integrated over all transcript isoforms per reference locus. The thin dashed red line indicates the line of equivalent expression. The thick dashed red lines indicate the 25% intervals. (TIF) [file pgen.1004365.s007.tif]

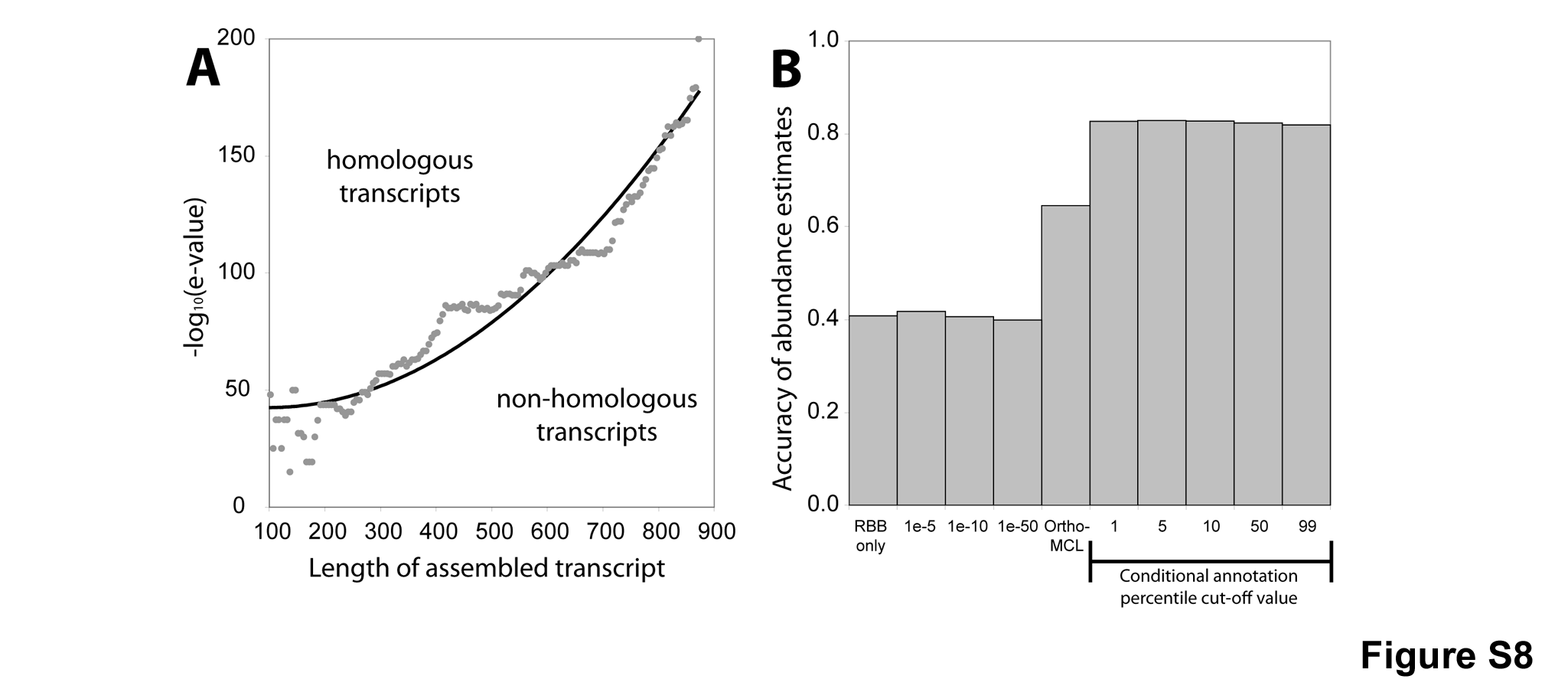

Supplement: Figure S8 — Example of conditional orthology assignment data fitting. (A) Grey dots indicate 1st percentile e-value of reciprocal best BLAST hits. Black line indicates the quadratic polynomial curve fit to the data. This line is used to identify putative homologues. Sequences of a given length that have e-values above the line are considered putative homologues. Those below the line are not. (B) The Spearman correlation in transcript abundances between the reference guided estimation and estimates generated using different transcript orthology assignment methods on the same de novo assembled transcriptome. “RBB only” means that only the reciprocal best BLAST transcripts were selected. E-value cut-offs (e.g. 1e-5) indicate the fixed value at which sequences were determined to be homologues. OrthoMCL indicates that OrthoMCL was used to cluster and identify orthologous transcript groups. Finally, the black bar indicates the effect of varying the percentile cut-off on the abundance estimate accuracy of the conditional orthology assignment method. (TIF) [file pgen.1004365.s008.tif]

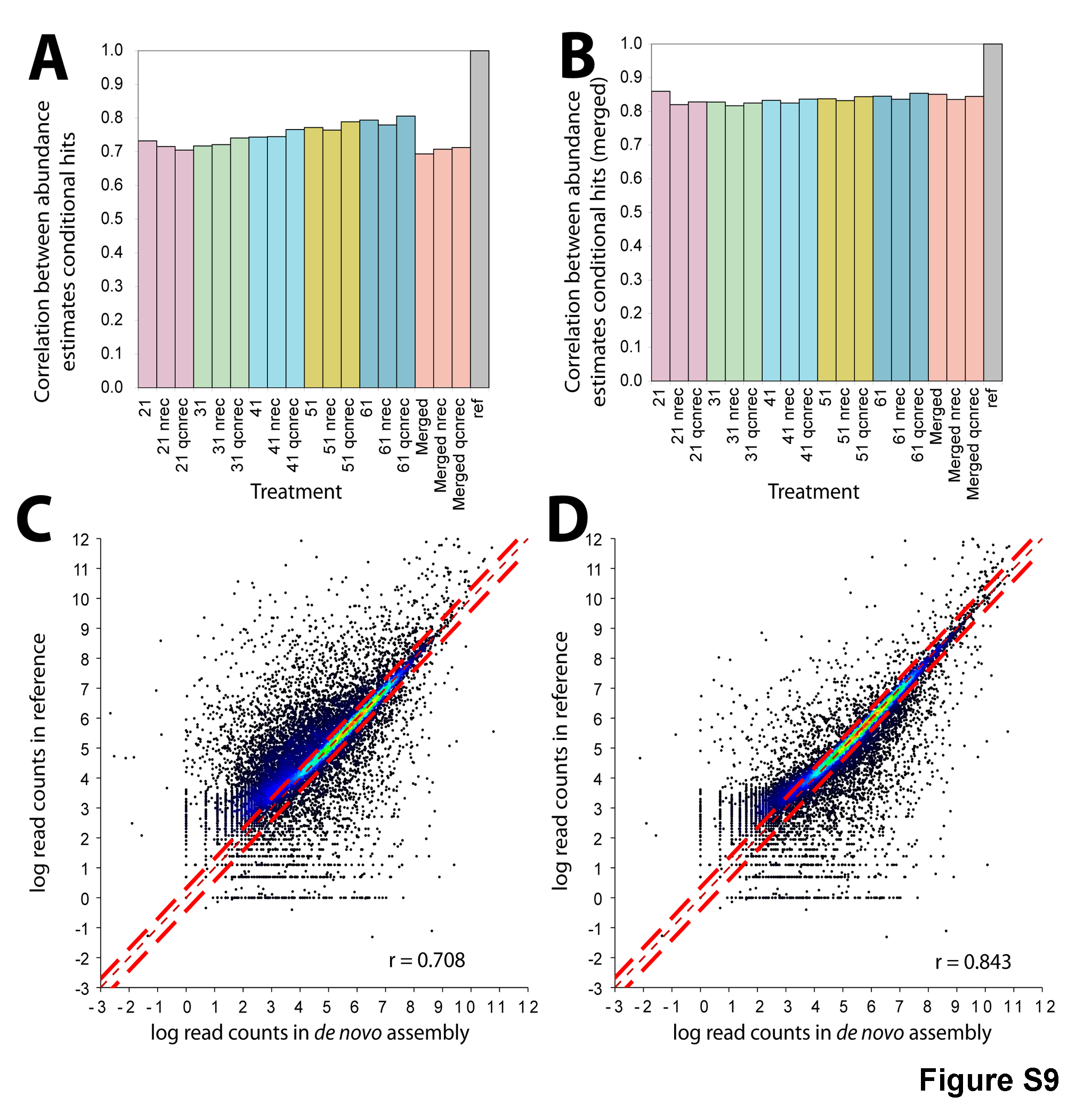

Supplement: Figure S9 — The effect of conditional orthology assignment on gene expression estimates. (A) Correlation in quantification derived from conditional assigned transcripts. (B) As in (A) but summed over all transcript isoforms per reference locus. For abbreviations see legend to Figure S4. (C) Scatter plot log transformed read counts obtained from RSEM quantification of the conditional assigned de novo assembly and the reference transcriptome (example shown is the merged non-redundant error corrected sample). (D) As in (C) but summed over all transcript isoforms per reference locus. The thin dashed red line indicates the line of equivalent expression. The thick dashed red lines indicate the 25% intervals. (TIF) [file pgen.1004365.s009.tif]

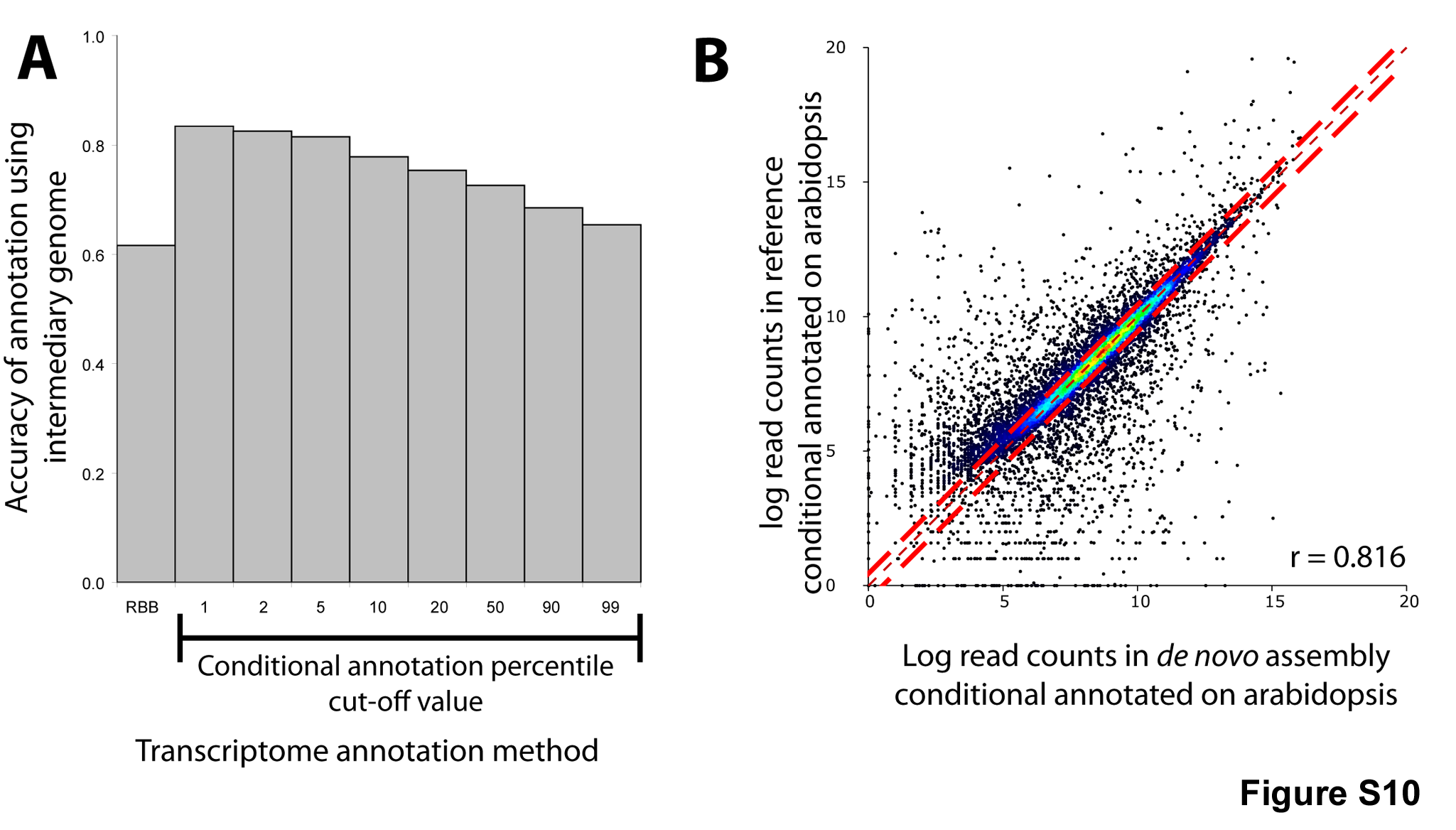

Supplement: Figure S10 — The effect of using an intermediary reference proteome to assign transcripts and compare expression data. (A) The effect of percentile cut-off on the homologue detection accuracy (F1 score) of the conditional assignment method. (B) Comparison between log transformed read counts in assembled and reference transcriptome using the Arabidopsis thaliana transcriptome as an assignment intermediary. The thin dashed red line indicates the line of equivalent expression. The thick dashed red lines indicate the 25% intervals. (TIF) [file pgen.1004365.s010.tif]

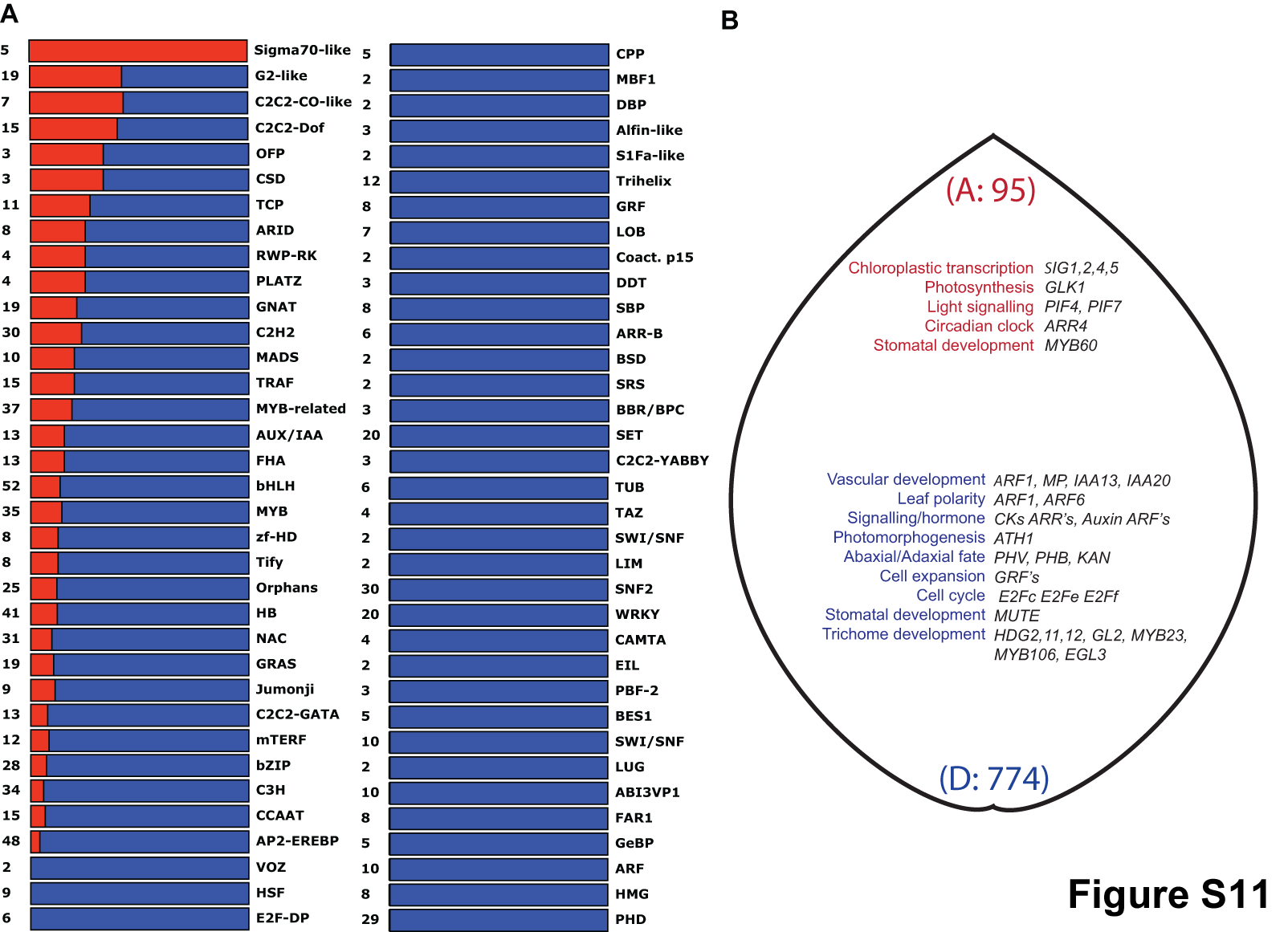

Supplement: Figure S11 — Behaviour of all transcription factor families identified within the C. gynandra leaf gradient. Transcription factors were classified by family (A) and the proportion of the genes ascending (red) or descending (blue) were assessed for each of the 70 families detected. A schematic leaf (B) shows specific TF families with related functions in function of their expression profile. (TIF) [file pgen.1004365.s011.tif]

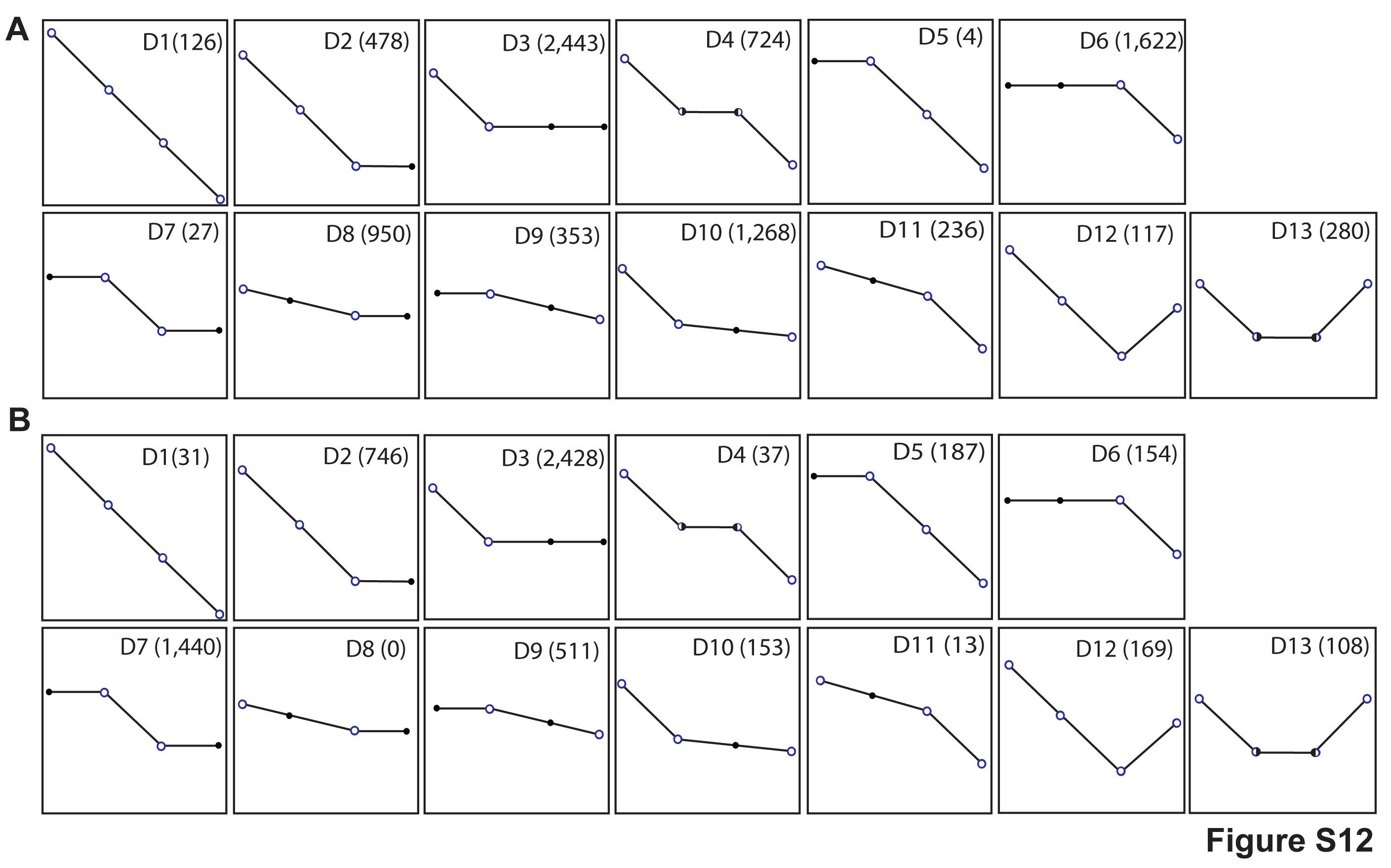

Supplement: Figure S12 — Classification of transcripts into thirteen descending behaviours as leaves of C. gynandra (A) and maize mature (B). Statistically significant differences between neighbouring tissue types are delineated by blue circles in descending filters, non-significant differences are indicated by black circles. The total number of genes that exhibit each behaviour is presented in parentheses. (TIF) [file pgen.1004365.s012.tif]

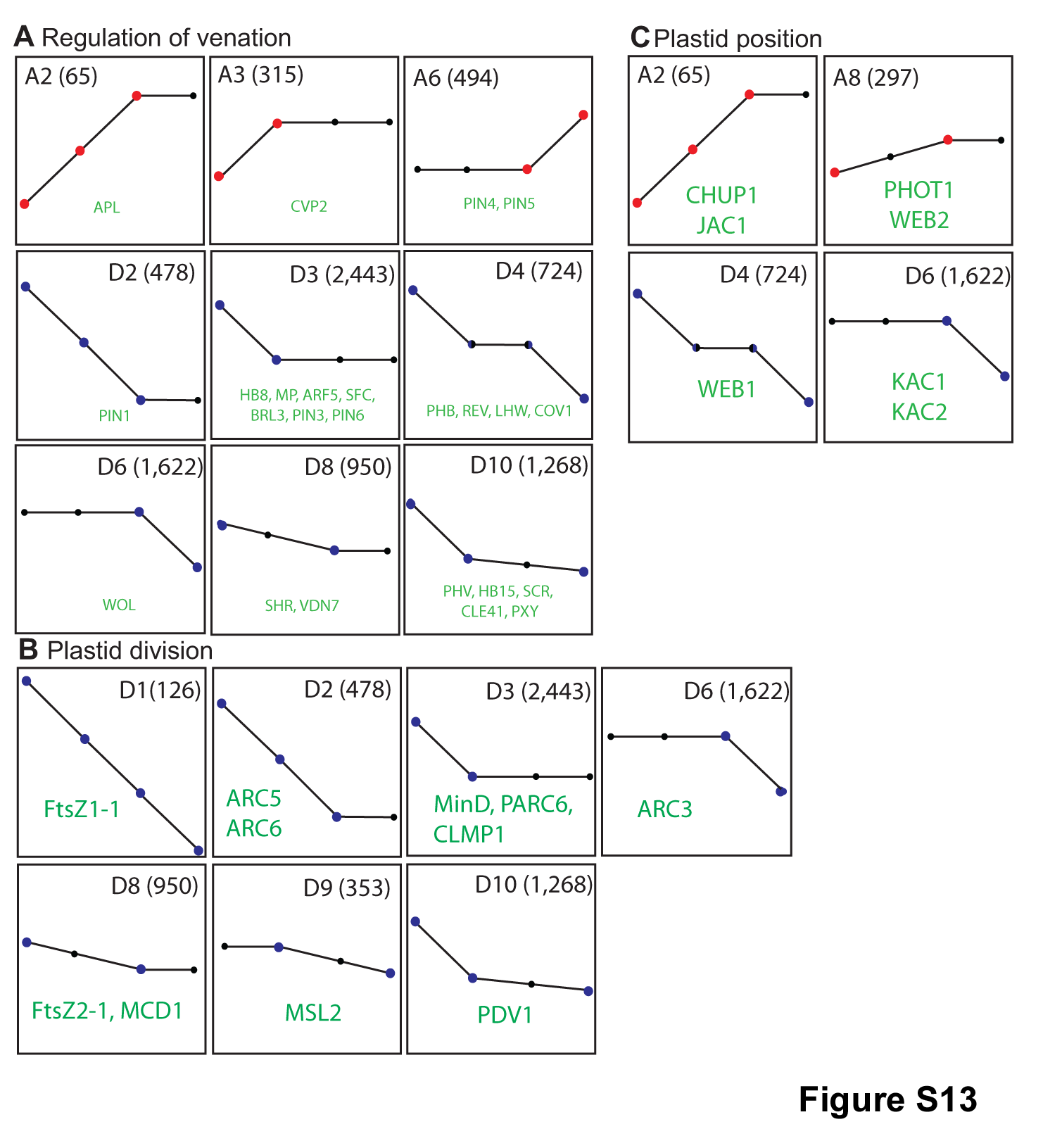

Supplement: Figure S13 — Behaviour of genes involved in chloroplast division, positioning and venation in the base, middle and tip of 3 mm leaves as well as mature leaves of C. gynandra. Genes annotated as being important for venation were found in two ascending and eight descending filters (A), genes for chloroplast positioning in two descending and two ascending filters (B), and those annotated as being involved in plastid division in seven descending clusters (C). The total number of genes in each filter is annotated in parentheses, and specific genes in each class depicted in green text. (TIF) [file pgen.1004365.s013.tif]

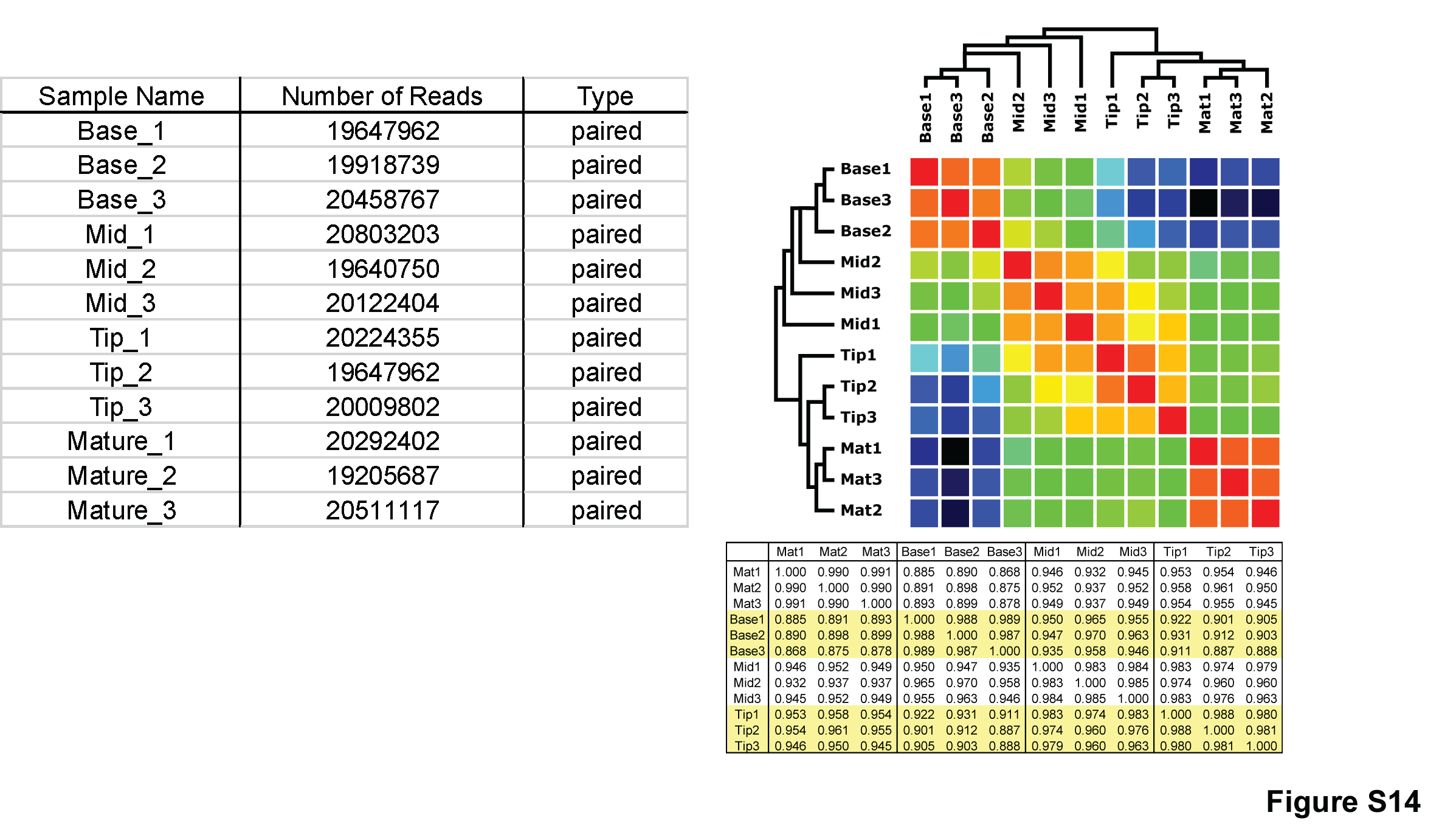

Supplement: Figure S14 — Spearman ranked correlation coefficients for pairwise sample comparisons of global mRNA abundance estimates. Correlation shown as a heatmap (strongest correlation in red, weakest correlation black) with numerical provided below. Triplicate sequencing replicates for each of the 4 tissue sections are shown. (TIF) [file pgen.1004365.s014.tif]
