# Supplementary material for: Deep Evolutionary Comparison of Gene Expression Identifies Parallel Recruitment of Trans-Factors in Two Independent Origins of C4 Photosynthesis
Source: PLoS Genet. 2014 Jun 5;10(6):e1004365. doi: 10.1371/journal.pgen.1004365 (PMC4046924; doi:10.1371/journal.pgen.1004365)
Supplement: Text S1 — Supplementary methods. (DOCX) [file pgen.1004365.s030.docx]

## Supplementary Methods

## Background information and motivation for methodology

High-throughput sequencing of DNA and RNA has revolutionised our ability to assess the genetic basis of many complex biological traits. For organisms that have sequenced and annotated genomes, short reads can be directly mapped to the genome and quantitative estimates of gene expression level (as well as splice variants and sequence differences) can be defined using a variety of different methods [[1-3](#_ENREF_1)]. Despite rapidly decreasing costs, genome sequencing is still beyond the reach of most research budgets and hence genome guided transcriptome analysis is not yet possible for most species. For example, within the land plants only ~0.005% of all extant plant species have genome resources available. Given this cost barrier, the sequencing of transcriptomes is an attractive alternative. However, the utility of any *de novo* transcriptome analysis is influenced by both the quality of the assembly and the accuracy of the orthology assignment of the assembled transcripts.

There are several algorithms that allow *de novo* transcriptomes to be assembled from high-throughput sequence data without a reference genome, popular amongst these are those based on de Bruijn graphs such as Trinity [[4](#_ENREF_4)], Trans-Abyss [[5](#_ENREF_5)] and velvet/oases [[6](#_ENREF_6),[7](#_ENREF_7)]. These methods have primarily focussed on maximising assembly of full-length transcripts [[4](#_ENREF_4)], detection of splice variants [[5](#_ENREF_5)] and the specificity and sensitivity of the assembled nucleotide sequences [[6](#_ENREF_6),[7](#_ENREF_7)]. Multiple post-assembly pipelines have also been produced to process the output of these assemblers to further increase the sensitivity and specificity of the assemblies [[5](#_ENREF_5),[8](#_ENREF_8)]. These post-assembly methods primarily achieve this increase in performance by combining assemblies generated using different assembly parameters. The ability of these methods to assemble full-length transcripts and detect splice variants has been extensively studied [[2](#_ENREF_2),[5](#_ENREF_5),[6](#_ENREF_6)] by comparing *de novo* and genome guided transcriptome assembly methods.

While there has been much focus on the improvement of methods to deal with issues which are specific for the assembly of transcriptomes, the improvement of orthology assignment methods which consider transcriptome specific properties and artefacts has received less attention. However, there are several well developed methods for orthology assignment from genome sequence that can be categorised into two different groups. Those which define homologous groups of sequences between two or more taxa by graph based clustering of BLAST [[9](#_ENREF_9)] similarity searches [[10-12](#_ENREF_10)], and those which analyse the results of BLAST similarity searches with phylogenetic inference methods [[13](#_ENREF_13),[14](#_ENREF_14)]. The accuracy of these (and other) methods has been extensively tested on whole genome and predicted proteome data yielding contradictory results [[15-17](#_ENREF_15)]. Though the results are contradictory, it is clear that both method groups perform well when sequences are present as single copy genes in the datasets being compared and perform less well when trying to distinguish highly similar sequence groups (such as multi-copy genes) from each other.

The issue of multiple-copy genes and near identical gene-groups is particularly relevant for the analysis of transcriptome data. It is to be expected that following *de novo* assemblies of RNAseq data, most gene loci will be represented by multiple assembled transcript variants [[2](#_ENREF_2),[5](#_ENREF_5),[6](#_ENREF_6)]. These transcripts may differ from each other in many ways, for example though single nucleotide polymorphisms, alternative splicing of internal exons, alternative terminal exons, incomplete assembly due to low sequence coverage and chimeric transcripts due to assembly errors. Thus methods which attempt to produce a one-to-one homology map between an assembled transcriptome and a reference genome will likely result in multiple missing orthology assignments in the *de novo* assembly. Therefore any method which is specifically designed for orthology assignment from *de novo* assembled transcriptomes should be able to deal with multiple different transcript variants for any given gene. Recent analyses indicate that one of the simplest orthologue detection methods (i.e. just using reciprocal best-BLAST) produces the fewest false positive orthology assignment errors [[17](#_ENREF_17)]. However, as reciprocal best-BLAST methods produce a one-to-one orthology map they are poorly suited for the analysis of transcriptome data and information would likely be lost due to the increased redundancy arising from multiple assembled transcript variants generated during assembly.

We therefore developed a novel supervised machine learning method which learns sequence similarity parameters for likely transcript variants from a pairwise reciprocal best-BLAST analysis and then uses this information to classify non-reciprocating BLAST hits as either homologous or non-homologous transcripts. The efficacy of this method was tested using paired-end Illumina RNAseq from *Oryza sativa* (rice). As the rice genome has been sequenced, the accuracy of the orthology assignment method can be directly assessed. A web-based implementation of the conditional orthology assignment method is freely available for use at **http://bioinformatics.plants.ox.ac.uk/annotate/**.

### Method summary

This method identifies homologous gene sequences by learning sequence similarity parameters for likely sequence homologues from a reciprocal best BLAST analysis. Thus the method accounts for both the global sequence divergence between the query sequence set and the reference sequence set and any bias in query sequence length caused by artefacts in the assembly process. **Figure S3** depicts an overview of the process.

### Implementation

To generate a set of reciprocal best BLAST (RBB) hits the full set of assembled transcripts is searched against the reference proteome using BLASTx and the reference proteome is searched against the assembled transcripts using tBLASTn (**Figure S3A**). The reciprocal best hits between these two datasets are identified (**Figure S3B**) and accepted as orthologues. These hits are then grouped according to the length of the assembled transcript. For each length group the RBB hits are ranked and an e-value of a selected percentile is recorded. A matrix of all e-values and mean lengths for each group is then fit to a quadratic polynomial model by least-squares fitting (**Figure S3C**). The function describing this curve is then used to classify all non-reciprocating transcripts of any given length; those above the curve are accepted as putative homologues and those below are rejected (**Figure S3D**). Thus orthology assignment is conditioned on the assembled transcript length and the global sequence divergence between the assembled and reference transcriptome (**Figure S3D**). As BLAST e-values have a floor of 1x10^-180^ any sequence producing an e-value of 1x10^-180^ or 0 are considered homologues. Due to this floor the curve is also terminated at 1x10^-180^ and sequences longer than the value at which this lower limit is reached must have e-values of 1x10^-180^ or 0 to be considered to be putative homologues. In summary, this orthology assignment method considers the global sequence divergence between the entire query and reference set and takes into account any bias in query sequence length which may arise from assembly artefacts. Therefore this method allows for orthology assignment and grouping of partially assembled sequences. Though full length assembled transcripts often contain UTR sequences, we used the total assembled transcript length rather than the hit length to prevent chimeric mis-assemblies from biasing the results due to a short, high scoring hit in a long mis-assembled transcript.

## Method validation

### Generation of a diverse set of *de novo* assemblies for method validation

To generate a set of test assemblies on which to test our orthology assignment method we sequenced and assembled 22.5 million 100bp paired end reads from random hexamer reverse-transcribed polyA enriched RNA from mature leaves of *Oryza sativa*. We set out to generate assemblies that differed from each other on several commonly used assembly statistics so that a realistic assessment of our orthology assignment method could be performed over a range of different assemblies. There are several algorithms that are capable of assembling transcriptomes from short read data. For the purposes of this analysis all assemblies were generated using Velvet/Oases [[6](#_ENREF_6)]. While any assembly method could have been selected, Velvet/Oases was chosen as it is fast, customisable, robust and has been shown to be equivalent to or improve significantly on other available *de novo* assemblers [[6](#_ENREF_6),[7](#_ENREF_7)]. To produce a set of test assemblies that varied in their properties multiple assemblies were generated using different assembly parameters. In addition to varying the assembly parameters multiple different pre and post assembly processing strategies were used. These comprise three read processing strategies 1) Raw sequence reads were used without any processing or filtering; 2) Reads were subjected to two cycles of read error correction using the ALLPATHS-LG [[18](#_ENREF_18)] read error correction tool and then filtered to remove all redundant read-pairs. 3) The same as in 2 but with an additional step whereby reads were trimmed from both the 5’ and 3’ ends based on a read phred quality threshold of >5 resulting in the removal of 12.1% of bases from the 22.5 million paired end reads. Assemblies were generated for a range of kmer values (k=21 to to k=61 inclusive) and these assemblies were then interrogated individually or subject to post assembly merging and redundant transcript removal using usearch [[19](#_ENREF_19)]. The resulting assemblies exhibit a spectrum of qualitatively and quantitatively different characteristics which are described in detail below. Each assembly was quantified using the uncorrected reads and the RSEM method [[1](#_ENREF_1)]. To provide a control for this analysis, the uncorrected and unfiltered reads were also mapped and quantified against the known rice transcriptome using RSEM [[1](#_ENREF_1)]. Transcripts from this genome guided rice transcriptome were selected as the reference set that should be assembled and accurately quantified by a *de novo* assembly approach. This combined dataset (reference and *de novo* assemblies) represents the raw data on which we tested the performance of the orthology assignment method.

### Quantitative and qualitative assessment of *de novo* assemblies prior to method validation

Read processing prior to assembly had a marked effect on the length of the longest observed transcript (**Figure S4A**) with read error correction producing marked increases in maximum transcript length for assemblies using k-mer sizes 41, 51 and 61 but mixed or negative effects on assemblies using k-mer sizes 21 and 31. This effect was also seen when analysing the mean length of all assembled transcripts (**Figure S4B**). Correction of read errors and removal of redundant read pairs increased the mean transcript length for assemblies using k-mer sizes 31, 41, 51 and 61 (**Figure S4B**), and with the exception of k-mer 21, further processing of reads by clipping poor quality bases produced a reduction in mean transcript length (**Figure S4B**). In all cases the mean transcript length was dramatically shorter than that observed in the expressed reference transcriptome (**Figure S4B**). There was also a general trend for assemblies made using longer k-mers to have shorter mean transcript length. The same effect was seen when analysing the median transcript length (**Figure S4C**). In agreement with what was observed for analysis of mean and median lengths both read processing and k-mer size had a marked effect on N50 length with all assemblies having overall shorter lengths than anticipated from the reference (**Figure S4D**). Analysis of the number of transcripts greater than 1kbp also reflected the trends described above. For k-mers 21, 31 and 41 read processing reduced both the number of long transcripts (**Figure S4E**) and the total number of transcripts observed (**Figure S4F**). It is clear that the overall number of transcripts from all assembly methods was far higher than the expected number of gene models from the genome **(Figure S4F)** but that this effect decreased with increasing k-mer size. From the above analyses, none of the assemblies generated transcriptomes with identical properties to the reference transcriptome (obtained as described above by selecting genes from the genome with non-zero expression values) and assemblies varied in number and length of assembled transcripts.

To further determine the quantitative and qualitative differences between the test assemblies a comparison was made between each assembly and the set of detected reference transcripts derived from the rice genome. Here, each set of assembled transcripts was compared to the reference set of rice transcripts above using BLAST [[9](#_ENREF_9)]. Figure 4A shows the mean number of hits passing an e-value threshold of 1x10^-5^ for each gene in the reference set. Here, if each transcript in the reference transcriptome had 8 hits in the assembly passing this e-value threshold then a value of 8 would be obtained. This analysis revealed that on average multiple good hits were found in all assembled transcriptomes for each gene in the reference **(Figure S5A)**. Assemblies that were generated using larger k-mer sizes reduced this redundancy and this was further improved by read processing prior to assembly (**Figure S5A**). Larger k-mer assemblies appeared to have values that are more similar to the reference transcriptome (**Figure S5A**). However, it was clear that as well as reducing redundancy, increasing k-mer size also reduced the number of reference transcripts which can be detected (**Figure S5B**). There was a 10% reduction in the number of transcripts that were observed between k-mer size 21 and 61. For both k-mer 51 and 61, removing read errors and redundant reads improved the coverage of the expected transcriptome (**Figure S5B**). However, for shorter k-mer sizes this effect is not seen. Moreover, while multiple assembled transcripts hit each reference transcript (**Figure S5A**) it appeared that the opposite was not true (**Figure S5C**). k-mer choice alone had little effect on the number of reference transcripts which hit each assembled transcript but a combination of longer k-mers and read error correction led to transcript redundancy values closer to those observed for the reference transcriptome (**Figure S5A**). Increasing k-mer size also decreased the proportion of assembled transcripts that did not have hits in the reference transcriptome (**Figure S5D**). It is interesting to note that application of read processing prior to assembly produced different effects on nearly all measures dependent on k-mer size.

It has previously been shown that merging/combining assemblies made using different k-mers is beneficial to assembly quality [[5](#_ENREF_5),[6](#_ENREF_6),[8](#_ENREF_8)]. In accordance with this, combining assemblies generated using multiple k-mer sizes followed by removal of transcripts that were ≥95% identical led to an increased coverage of the reference transcriptome (**Figure S5B**) but incurred a cost of increasing redundancy (**Figure S5A**). The reason for this is apparent from a comparative analysis of genes that were detected by each assembly method (**Figure S6**). Here, the majority of transcripts are detectable in all assemblies (60%-61%, **Figure S6** centre of Venn diagrams) with each assembly containing small numbers of unique reference transcripts (0.5% **Figure S6C**:k51 - 3.2% **Figure S6A**:k21) not detected by other methods. The general trend observed was that application of read error correction increased the overlap between most assembly methods and decreased the number of transcripts which were unique to a given k-mer size. This demonstrates that (overall) read error correction does produce an improvement in assembly consistency. However, it is worth noting that read error correction also decreased the total number of transcripts which were be detected by ~1.9% (**Figure S6B&C**) with further reductions occurring from read clipping (**Figure S5C&6C**). In summary, merged assemblies generated from multiple different k-mer sizes increases reference transcript detection by >6% compared to single a k-mer assembly but incurred an increase in redundancy.

### The effect of orthology assignment method on gene expression quantification accuracy

As the purpose of our study is to compare gene expression data across species we sought to determine the effect of orthology assignment method on downstream quantification accuracy. For each assembly transcript abundance estimates were generated using RSEM [[1](#_ENREF_1)] and the accuracy of the abundance estimates was determined by comparing the estimates for each transcript generated by *de novo* assembly with those obtained using for the corresponding transcript in the reference transcriptome. To provide a baseline benchmark against which all orthology assignment methods can be compared we selected the set of reciprocal best BLAST (RBB) hits in each *de novo* assembly. For each assembly a set of RBB transcripts was identified by BLASTing the assembled transcriptome against the reference set and selecting those transcripts that are a bi-directional best hit. A Spearman’s ranked correlation coefficient was then determined for each pairwise comparison of abundance estimates obtained from the assembled transcripts and the reference genome. In all cases only those genes that had positive expression values in either the assembly or the reference were used to compute the correlation values. This prevents over-estimation of correlation by inclusion of multiple non-expressed genes. This also penalizes the correlation for false positive (incorrect orthology assignment) and false negative (missing orthology assignment) errors. Thus global expression level correlation represents a stringent, conservative and holistic measure of the utility of an orthology assignment method for expression level comparison.

Comparing the abundance estimates between each of the *de novo* assemblies and the reference transcriptome revealed that all methods achieved correlation coefficients of ~0.3 when considering RBB hits alone (**Figure S7A & C**). This value increases to ~0.4 when summing abundance estimates at the gene locus level (**Figure S7B & D**). There is no obvious systematic bias occurring in the assembled transcriptome as estimates derived from assembled transcripts can be either higher or lower than expected over multiple orders of magnitude (**Figure S7C & D**).

Critically, the correlation values observed in the above tests (**Figure S7**) are low. This low correlation concomitantly reduces the power of any comparative gene expression analysis and hence there is both scope and need to increase the orthology assignment performance. As our data indicate that assembled transcriptomes have increased redundancy (**Figure S5A**) and that summing transcript abundances at the locus level increases the accuracy of the comparison we developed a novel supervised machine learning method to facilitate improved orthology assignment and grouping of transcript variants. Importantly this method takes into account the bias in length of the assembled transcripts which varies depending on assembly method (**Figure S4**) and the global sequence divergence between the two datasets being compared (which varies depending on the evolutionary distance between species).

### Transcript orthology assignment by conditional BLAST improves accuracy of abundance estimates

Our novel orthology assignment method classifies sequences as homologues or not homologues by learning from a RBB analysis between two transcriptomes. The method starts by identifying the set of RBB hits between a *de novo* assembly and a reference transcriptome. The hits are grouped according to the length of the assembled transcript and for each length group the RBB hits are ranked by e-value. A percentile cut-off is selected and the e-value of that percentile is recorded. A matrix of all e-values and mean lengths for each group is then fit to a quadratic polynomial model by least-squares fitting (**Figure S3C & 8A**). The function describing this curve is then used to define a unique e-value cut-off for each assembled transcript that is thus dependent on the length of the assembled transcript and the global sequence divergence between the assembled and reference transcriptome (**Figure S3C & 8A**). A range of percentile cut-offs were interrogated and the accuracy of the resulting abundance estimates was determined (**Figure S8B**). In all cases the accuracy of the conditional orthology assignment set was approximately twice as high as that obtained using RBB alone (**Figure S8B**). Moreover, the conditional orthology assignment method also outperformed a simple method which used a set e-value cut-off for identifying and grouping homologous transcripts as well as more complex methods such as OrthoMCL (**Figure S8B**). Also of note is the number of transcripts which are identified by each method. In this instance RBB identified 31,299 transcripts as putative homologues of the reference set, OrthoMCL identified 57,079 and our conditional BLAST method identified 76,598 (of the 87,235 transcripts present in the merged assembly).

For all k-mer sizes and read processing strategies inclusion of conditional BLAST assigned transcripts dramatically increased the correlation in gene expression between the *de novo* assemblies and the reference (**Figure S9A & C**). A further marked improvement in abundance estimate accuracy was seen when abundances were summed for each locus in the reference genome. Here, all k-mer sizes and processing steps achieving a Spearman’s ranked correlation between 0.82-0.86 (**Figure S9B& D**). This increase in correlation represents a ~2.7 fold increase in abundance estimate accuracy over conventional RBB or set e-value cut-off based orthology assignment. Importantly, merged assemblies also achieve the same high correlation scores demonstrating that the increased redundancy incurred by merging assemblies is negated by the conditional orthology assignment method and thus merging assemblies is a valid approach for capturing the maximum amount of information present in *de novo* analyses.

### Orthology assignment using an intermediary reference genome

In the above analyses the *de novo* assemblies are being compared to a genome guided transcriptome for the same species. To simulate a comparison between two distantly-related non-model species and determine the incurred loss in detection and quantification accuracy that would occur from using a distantly related intermediary reference genome for transcript orthology assignment, we utilised the non-redundant predicted proteome of *Arabidopsis thaliana* as a reference against which we performed orthology assignment using our conditional method on both the *de novo* and genome guided transcriptomes of rice. Arabidopsis was selected as it has a well annotated genome and transcriptome (http://www.arabidopsis.org/) and is distantly related to rice [[20](#_ENREF_20)] having diverged 150-200 million years ago. Previous comparative analysis of highly conserved rice and arabidopsis sequences revealed that on average highly conserved nucleotide sequences are ~55% identical between the two species [[21](#_ENREF_21)], thus this represents a stringent and conservative test of the conditional orthology assignment method. Arabidopsis was also selected as its evolutionary distance to rice is equivalent to the evolutionary distance between *Cleome gynandra* and maize. Here the accuracy of the tested assignment methods is measured by the homologue detection accuracy (F_1_ score, **Figure S10A**). This analysis shows that using the conditional orthology assignment method and setting the length and composition conditioned e-value cut-off at the 1st percentile is the most accurate method for comparing transcriptomes via a distantly related intermediary reference transcriptome. This achieves a homologue detection accuracy of 0.829 (**Figure S10A**) and an abundance estimate accuracy of 0.816 (**Figure S10B**). When compared to assigning orthology for transcripts using the correct reference genome this represents a reduction in accuracy of ~2.9% (**Figure S9B & D**, r = 0.843). While orthology assignment via an intermediate genome is less accurate than orthology assignment using the correct reference genome, it is considerably more accurate than orthology assignment using conventional BLAST (**Figure S8B**, r = ~0.4) approaches or OrthoMCL (**Figure S8B**, r = 0.649) and the correct reference genome and hence represents a significant improvement to *de novo* assembly analysis. Moreover, this high level of accuracy renders this method appropriate for comparative gene expression analyses across multiple species lacking reference genomes even over large evolutionary distances.

## Supplementary references

1. Li B, Dewey CN (2011) RSEM: accurate transcript quantification from RNA-Seq data with or without a reference genome. BMC Bioinformatics 12: 323.

2. Martin JA, Wang Z (2011) Next-generation transcriptome assembly. Nat Rev Genet 12: 671-682.

3. Trapnell C, Roberts A, Goff L, Pertea G, Kim D, et al. (2012) Differential gene and transcript expression analysis of RNA-seq experiments with TopHat and Cufflinks. Nat Protoc 7: 562-578.

4. Grabherr MG, Haas BJ, Yassour M, Levin JZ, Thompson DA, et al. (2011) Full-length transcriptome assembly from RNA-Seq data without a reference genome. Nat Biotechnol 29: 644-652.

5. Robertson G, Schein J, Chiu R, Corbett R, Field M, et al. (2010) De novo assembly and analysis of RNA-seq data. Nat Methods 7: 909-912.

6. Schulz MH, Zerbino DR, Vingron M, Birney E (2012) Oases: robust de novo RNA-seq assembly across the dynamic range of expression levels. Bioinformatics 28: 1086-1092.

7. Zerbino DR, Birney E (2008) Velvet: algorithms for de novo short read assembly using de Bruijn graphs. Genome Res 18: 821-829.

8. Surget-Groba Y, Montoya-Burgos JI (2010) Optimization of de novo transcriptome assembly from next-generation sequencing data. Genome Res 20: 1432-1440.

9. Altschul SF, Madden TL, Schaffer AA, Zhang J, Zhang Z, et al. (1997) Gapped BLAST and PSI-BLAST: a new generation of protein database search programs. Nucleic Acids Res 25: 3389-3402.

10. Dessimoz C, Boeckmann B, Roth AC, Gonnet GH (2006) Detecting non-orthology in the COGs database and other approaches grouping orthologs using genome-specific best hits. Nucleic Acids Res 34: 3309-3316.

11. Li L, Stoeckert CJ, Jr., Roos DS (2003) OrthoMCL: identification of ortholog groups for eukaryotic genomes. Genome Res 13: 2178-2189.

12. Remm M, Storm CE, Sonnhammer EL (2001) Automatic clustering of orthologs and in-paralogs from pairwise species comparisons. J Mol Biol 314: 1041-1052.

13. Chiu JC, Lee EK, Egan MG, Sarkar IN, Coruzzi GM, et al. (2006) OrthologID: automation of genome-scale ortholog identification within a parsimony framework. Bioinformatics 22: 699-707.

14. Wapinski I, Pfeffer A, Friedman N, Regev A (2007) Automatic genome-wide reconstruction of phylogenetic gene trees. Bioinformatics 23: i549-558.

15. Altenhoff AM, Dessimoz C (2009) Phylogenetic and functional assessment of orthologs inference projects and methods. PLoS Comput Biol 5: e1000262.

16. Chen F, Mackey AJ, Vermunt JK, Roos DS (2007) Assessing performance of orthology detection strategies applied to eukaryotic genomes. PLoS One 2: e383.

17. Salichos L, Rokas A (2011) Evaluating ortholog prediction algorithms in a yeast model clade. PLoS One 6: e18755.

18. Maccallum I, Przybylski D, Gnerre S, Burton J, Shlyakhter I, et al. (2009) ALLPATHS 2: small genomes assembled accurately and with high continuity from short paired reads. Genome Biol 10: R103.

19. Edgar RC (2010) Search and clustering orders of magnitude faster than BLAST. Bioinformatics 26: 2460-2461.

20. The Angiosperm Phylogeny G (2009) An update of the Angiosperm Phylogeny Group classification for the orders and families of flowering plants: APG III. Botanical Journal of the Linnean Society 161: 105-121.

21. Ewing R, Poirot O, Claverie JM (1999) Comparative analysis of the Arabidopsis and rice expressed sequence tag (EST) sets. In Silico Biol 1: 197-213.
